# Supplementary material for: Sequential MCR via Staudinger/Aza-Wittig versus Cycloaddition Reaction to Access Diversely Functionalized 1-Amino-1H-Imidazole-2(3H)-Thiones
Source: Molecules. 2019 Oct 21;24(20):3785. doi: 10.3390/molecules24203785 (PMC6832714; doi:10.3390/molecules24203785)
Supplement: Supplementary file 1 [file molecules-24-03785-s001.pdf]

Supplementary Material

for

Sequential MCR *via* Staudinger/aza-Wittig *versus* cycloaddition reaction to access diversely functionalized 1-amino-1*H*-imidazole-2(3*H*)-thiones

Cecilia Ciccolini, Giacomo Mari, Gianfranco Favi *, Fabio Mantellini, Lucia De Crescentini and Stefania Santeusanio *

Department of Biomolecular Sciences, Section of Chemistry and Pharmaceutical Technologies, University of Urbino "Carlo Bo", Via I Maggetti 24, 61029 Urbino (PU), Italy; c.ciccolini@campus.uniurb.it (C.C.), g.mari@campus.uniurb.it (G.M.), fabio.mantellini@uniurb.it (F.M.), lucia.decrescentini@uniurb.it (L.D.C.)

* Correspondence: gianfranco.favi@uniurb.it (G.F.), stefania.santeusanio@uniurb.it (S.S.)

Table of contents

1. Synthetic procedure referred to in Scheme 2. (**S2)**
2. Copies of ^{1}H-NMR and ^{13}C-NMR spectra of compounds **II**, **2a**, **3a**, **5a–k**, **7a–c,** and **8a–c. (S3–S24)**
3. References. (**S2)**

Procedure referred to in Scheme 2

To a stirred solution of 2-chloro-*N*,*N*-dimethyl-3-oxobutanamide (**A**) (1 mmol) in AcOH (4 mL), finely crashed potassium thiocyanate (**B**) (1.5 mmol) was added at room temperature. After 1 hour, *tert*-butyl hydrazinecarboxylate (**C**) (1 mmol) was added portionwise and quickly. In a short time, the reaction mixture turned yellow and was left to stir until the disappearance of **A** (TLC check, 2 h). The crude reaction mixture was then treated with a diluted solution of 5% NaOH until neutrality, extracted with EtOAc (50 mL), and washed with water (3 × 10 mL). The combined organic phases were dried (Na_2SO_4), concentrated under reduced pressure, and purified by column chromatography eluting with cyclohexane:EtOAc mixture (from 1:1 to pure EtOAc) to afford **II** as major product.

tert-Butyl (5-((dimethylamino)carbonyl)-2-imino-4-methyl-1,3-thiazol-3(2*H*)-yl)carbamate (**II**): Yield 48% (144.2 mg) white powder from EtOAc / light petroleum ether; Mp 157–161 °C (dec.); ^{1}H-NMR (400 MHz, DMSO-d_6) δ 1.34 and 1.43 (2 s, 9 H, OBu^t), 1.90 (s, 3 H, CH_3), 2.94 [s, 6 H, $N(CH_3)_2$], 8.21 (br s, 1 H, NH), 9.48 (br s, 1 H, NH); ^{13}C-NMR (100 MHz DMSO-d_6) δ 12.7, 27.6, 27.8, 36.7, 80.5, 98.5, 137.0, 154.6, 158.0, 162.8; IR (Nujol, ν, cm^{-1}): 3320, 3254, 3200, 1736, 1633, 1607; MS *m*/*z* (ESI): 301.15 (M + H)$^+$; calcd. for $C_{12}H_{20}N_4O_3S$ (300.38): C, 47.98; H, 6.71; N, 18.65; found: C, 48.07; H, 6.75; N, 18.69 [1].

The partition of some signals here, as well as in the following cases, is caused by the N1-amide rotameric effect [2].

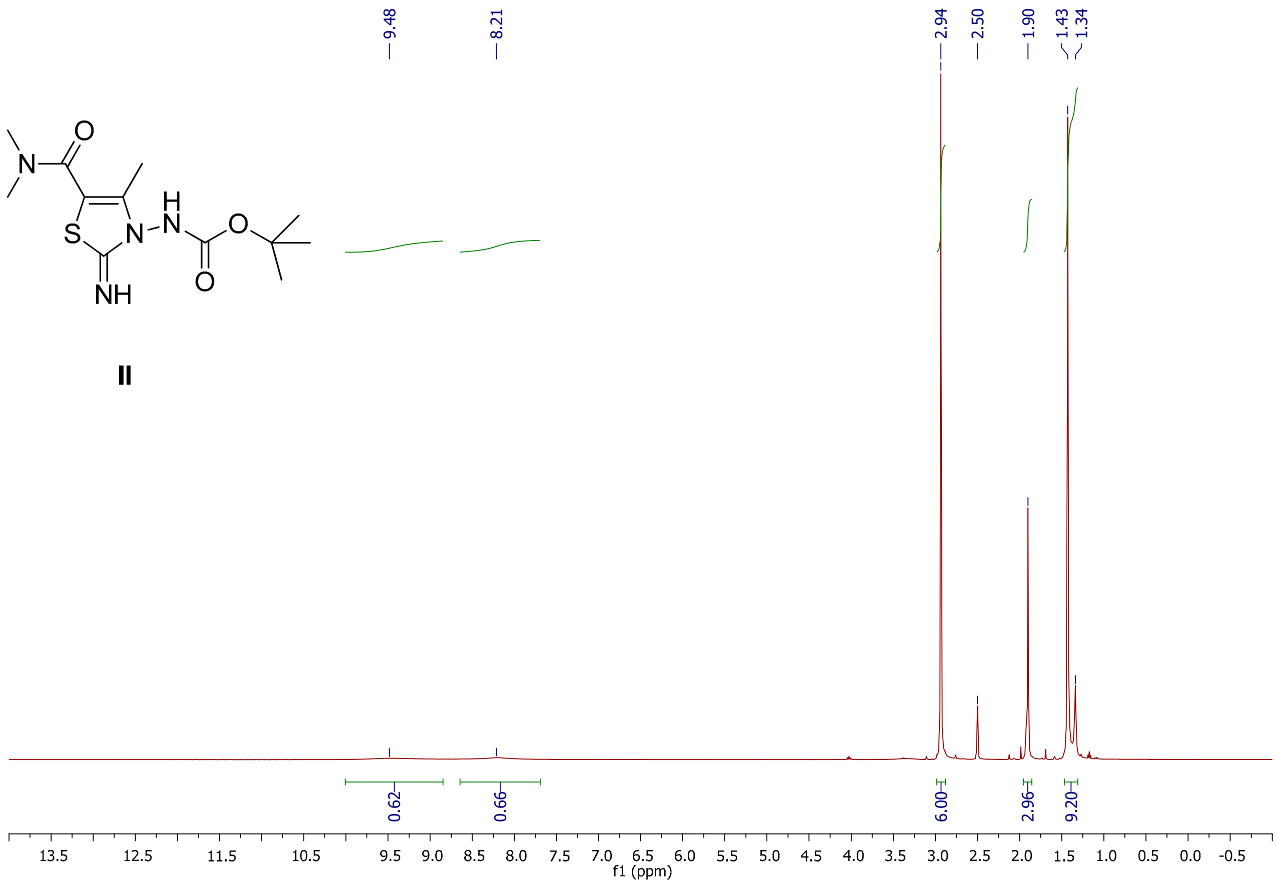

9.48
8.21
2.94
2.50
1.90
1.43
1.34
II
0.62
0.66
6.00
2.96
9.20
13.5 12.5 11.5 10.5 9.5 9.0 8.5 8.0 7.5 7.0 6.5 6.0 5.5 5.0 4.5 4.0 3.5 3.0 2.5 2.0 1.5 1.0 0.5 0.0 -0.5
f1 (ppm)

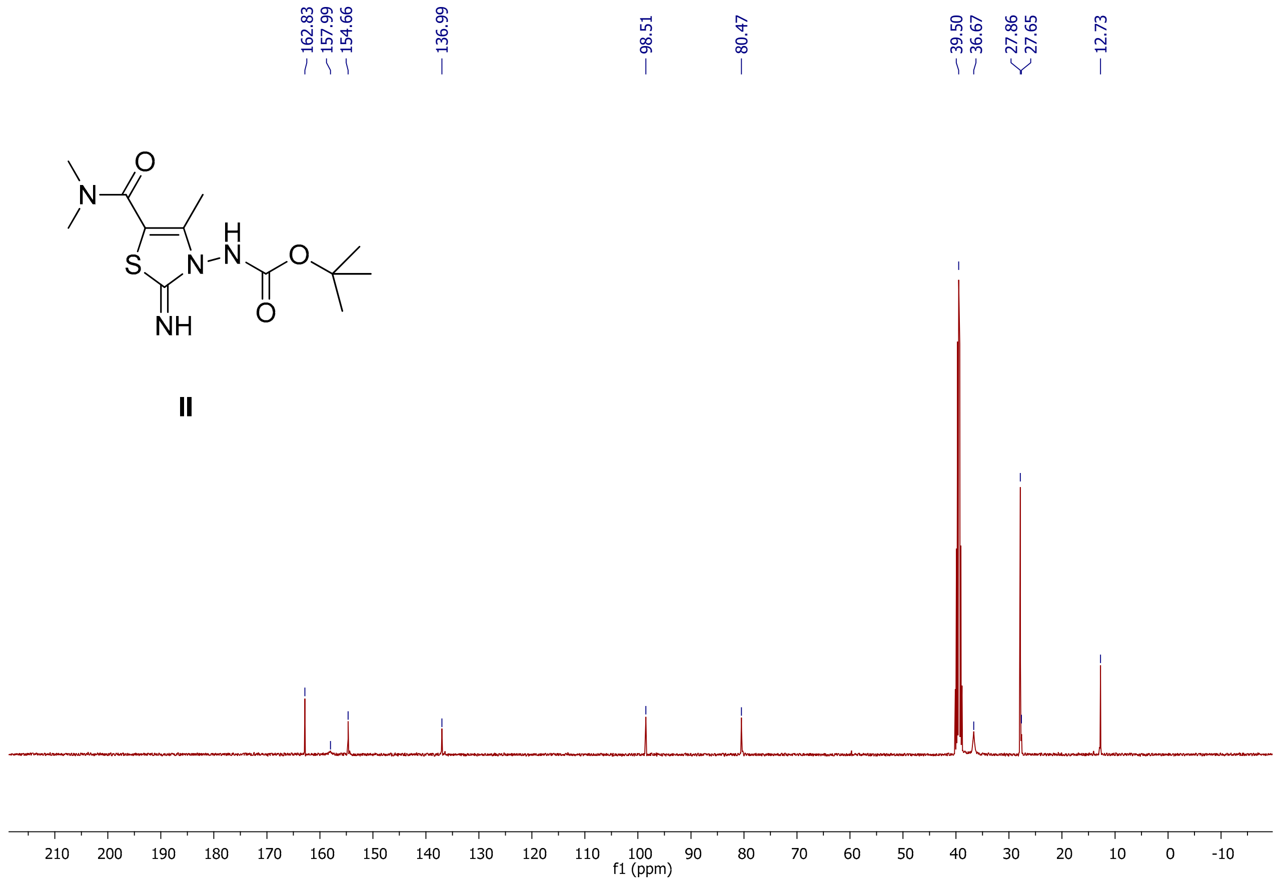

162.83
157.99
154.66
136.99
98.51
80.47
39.50
36.67
27.86
27.65
12.73
II
210 200 190 180 170 160 150 140 130 120 110 100 90 80 70 60 50 40 30 20 10 0 -10
f1 (ppm)

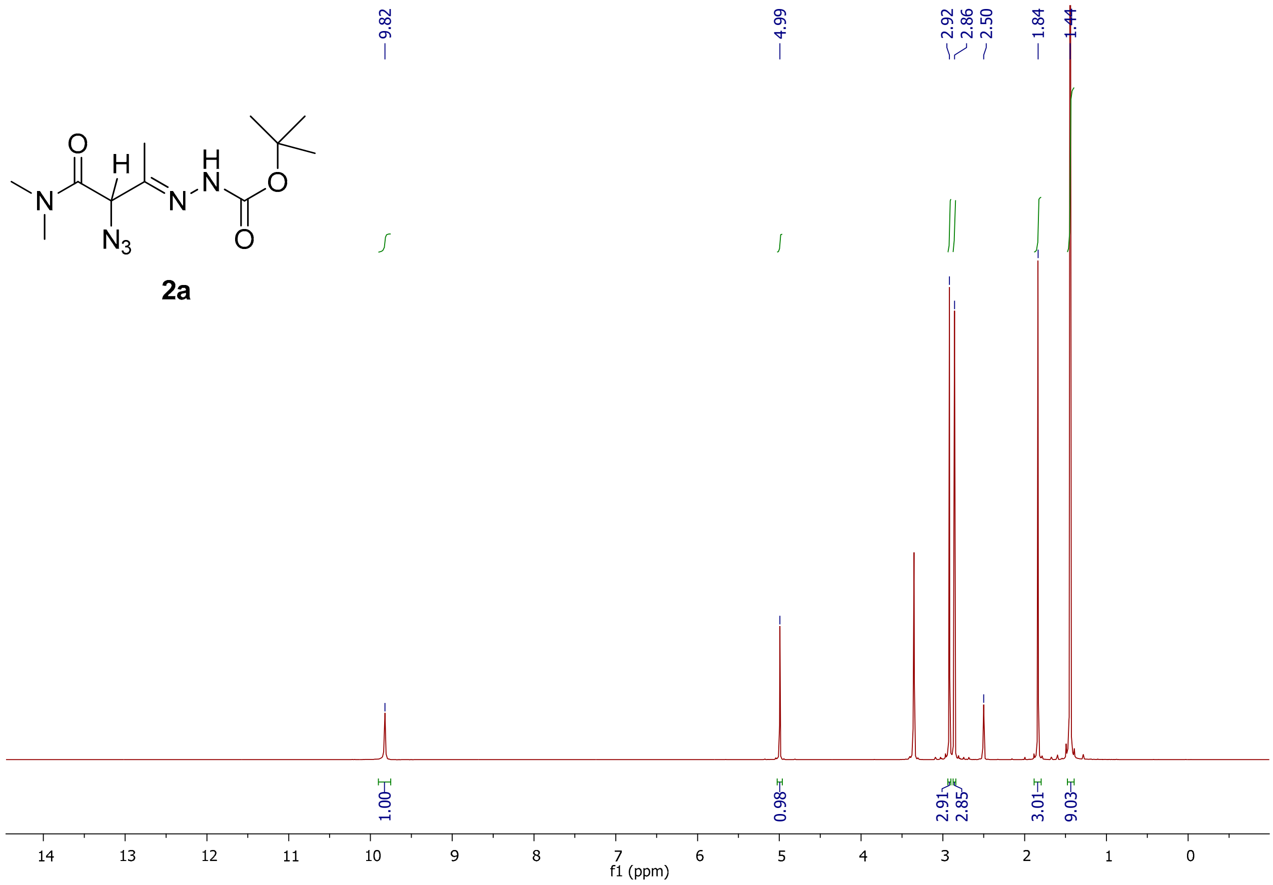
9.82
4.99
2.92
2.86
2.50
1.84
1.44
2a
1.00
0.98
2.91
2.85
3.01
9.03
14 13 12 11 10 9 8 7 6 5 4 3 2 1 0
f1 (ppm)

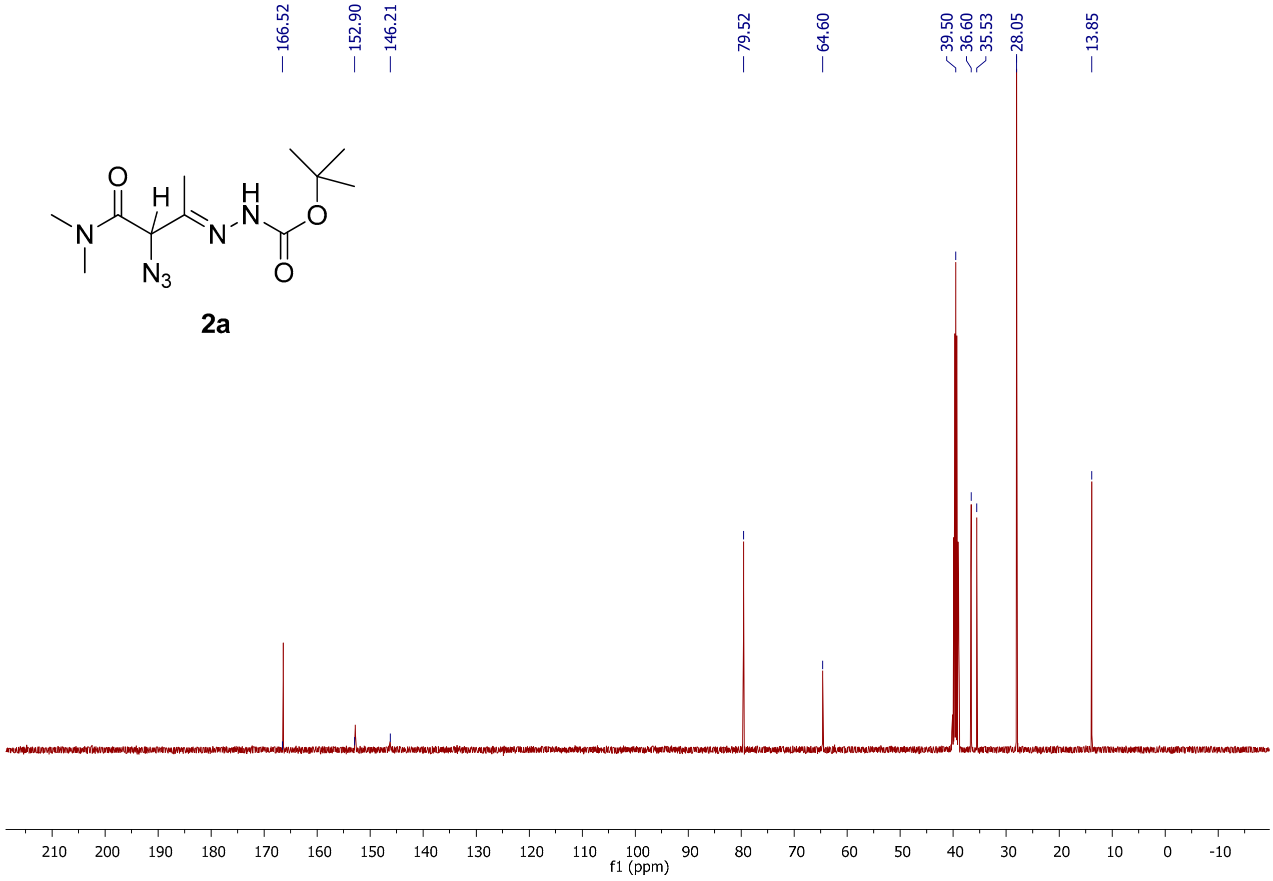
166.52
152.90
146.21
79.52
64.60
39.50
36.60
35.53
28.05
13.85
2a
210 200 190 180 170 160 150 140 130 120 110 100 90 80 70 60 50 40 30 20 10 0 -10
f1 (ppm)

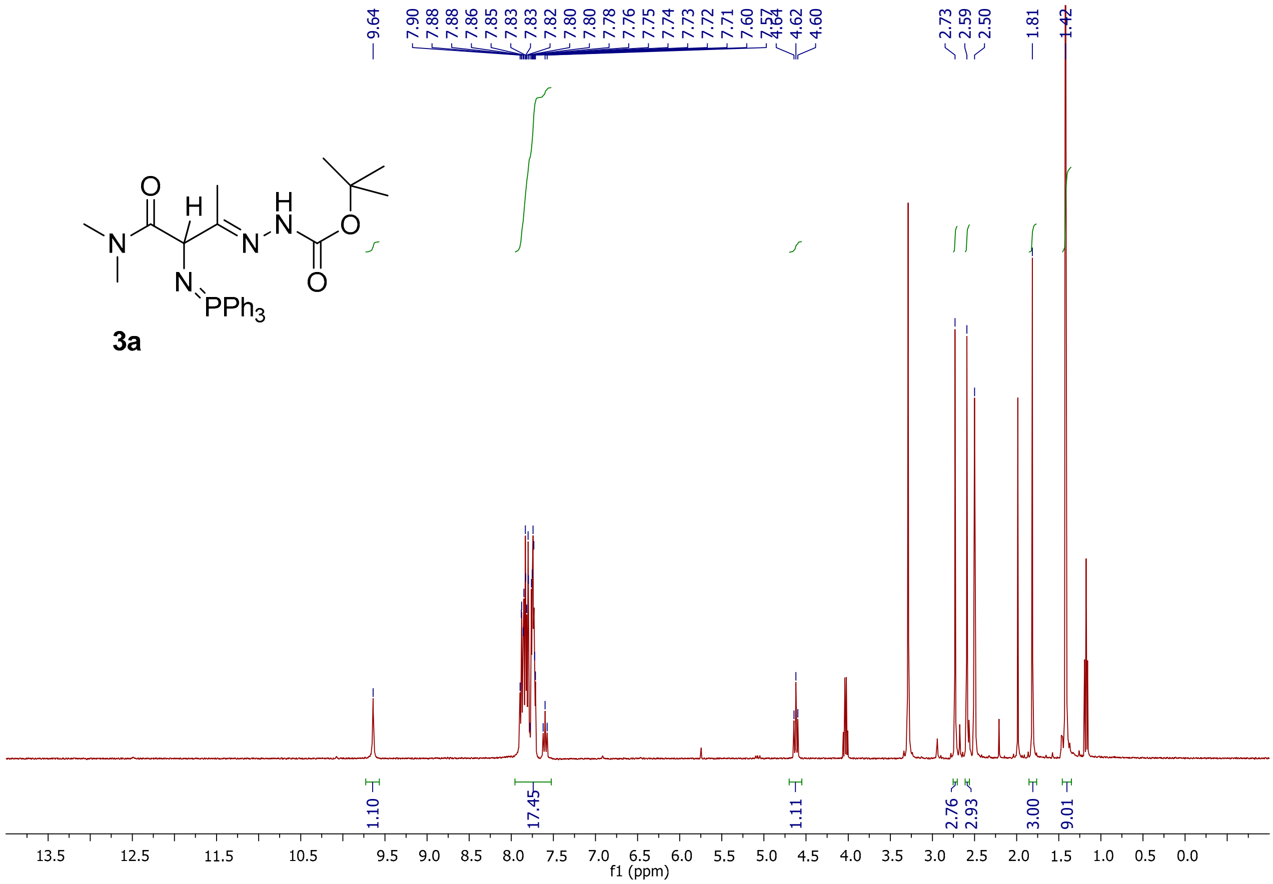
9.64
7.90 7.88 7.88 7.86 7.85 7.83 7.83 7.82 7.80 7.80 7.78 7.76 7.75 7.74 7.73 7.72 7.71 7.60 7.57
4.64 4.62 4.60
2.73 2.59 2.50
1.81
1.42
1.10 17.45 1.11 2.76 2.93 3.00 9.01
f1 (ppm)
3a

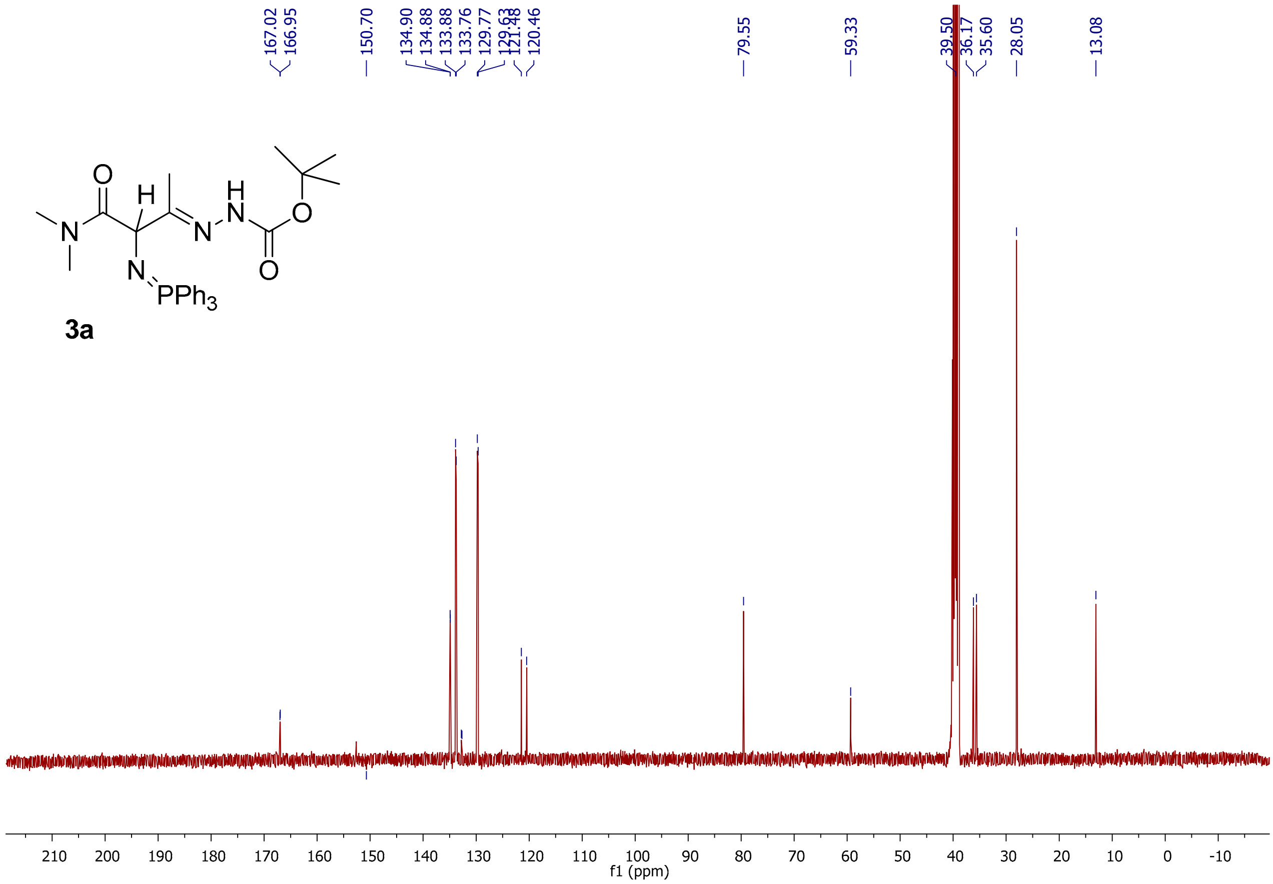
167.02 166.95
150.70
134.90 134.88 133.88 133.76 129.77 129.63 121.48 120.46
79.55
59.33
39.50 36.17 35.60
28.05
13.08
f1 (ppm)
3a

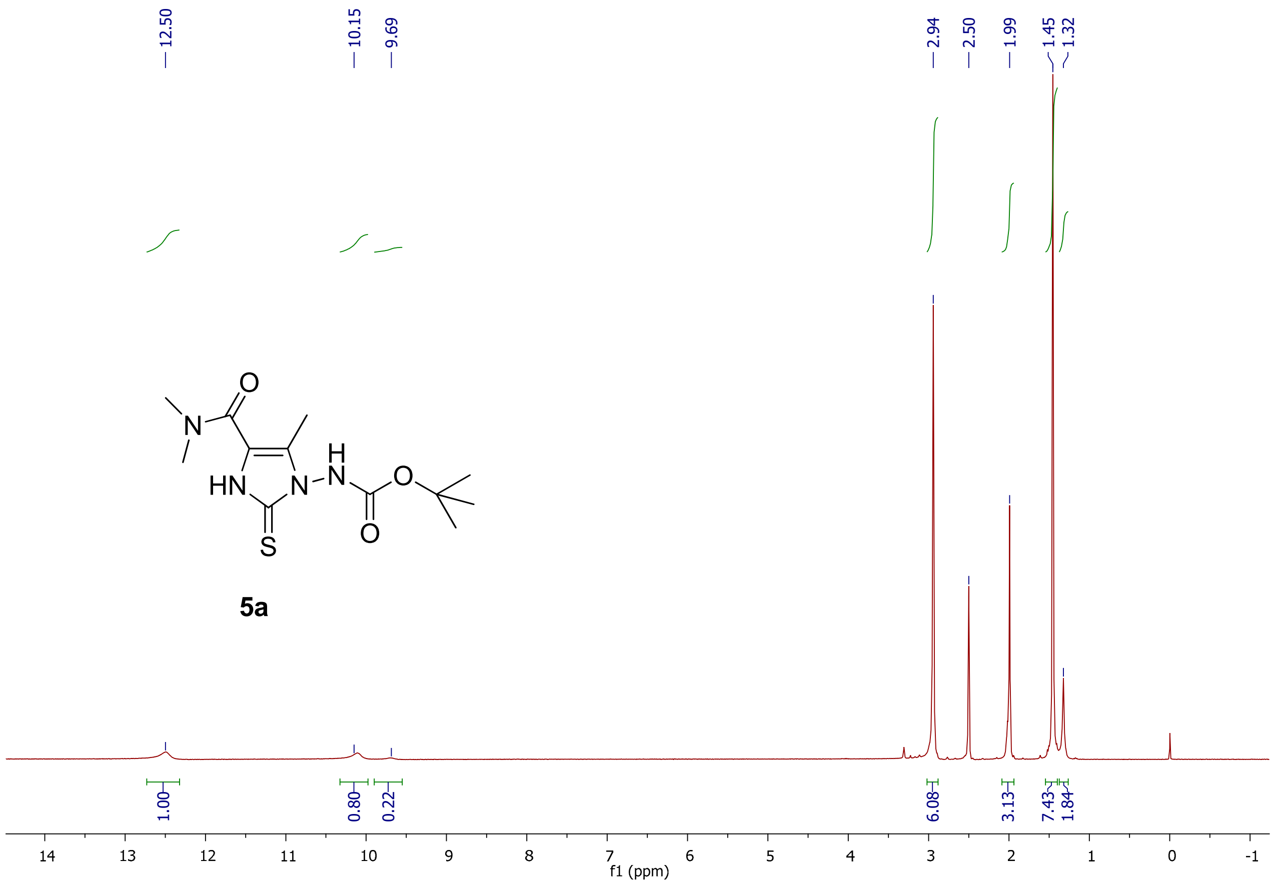
12.50
10.15
9.69
2.94
2.50
1.99
1.45
1.32
5a
1.00
0.80
0.22
6.08
3.13
7.43
1.84
f1 (ppm)

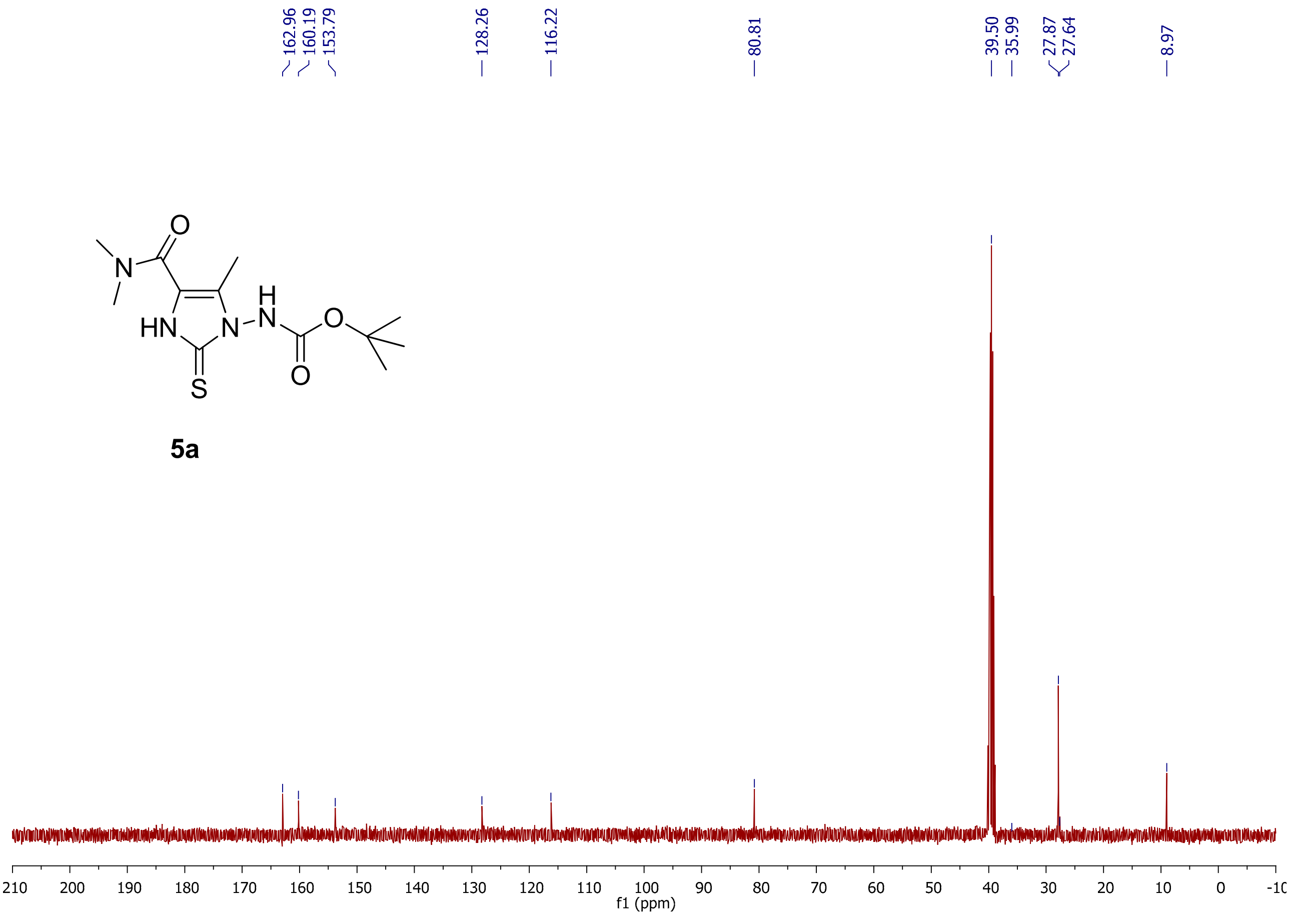
162.96
160.19
153.79
128.26
116.22
80.81
39.50
35.99
27.87
27.64
8.97
5a
f1 (ppm)

HMQC 5a

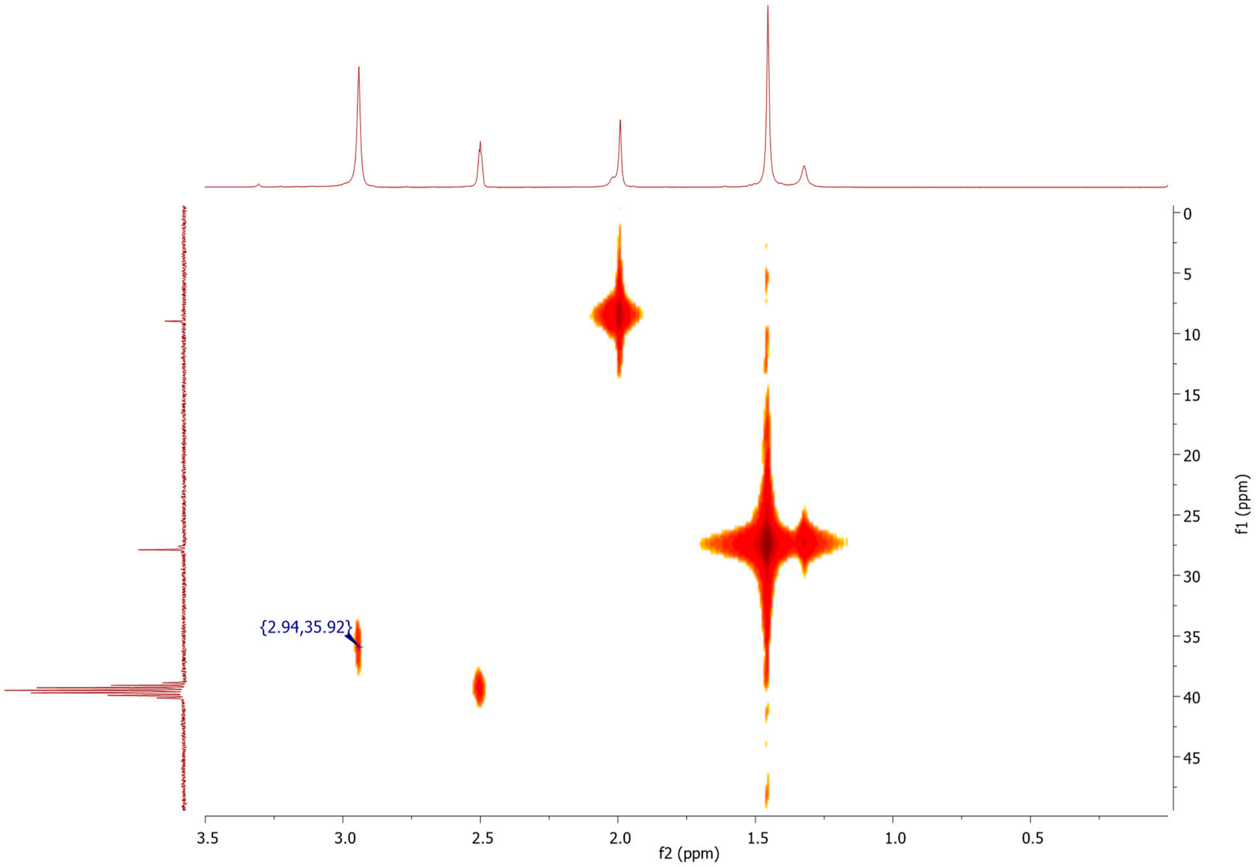

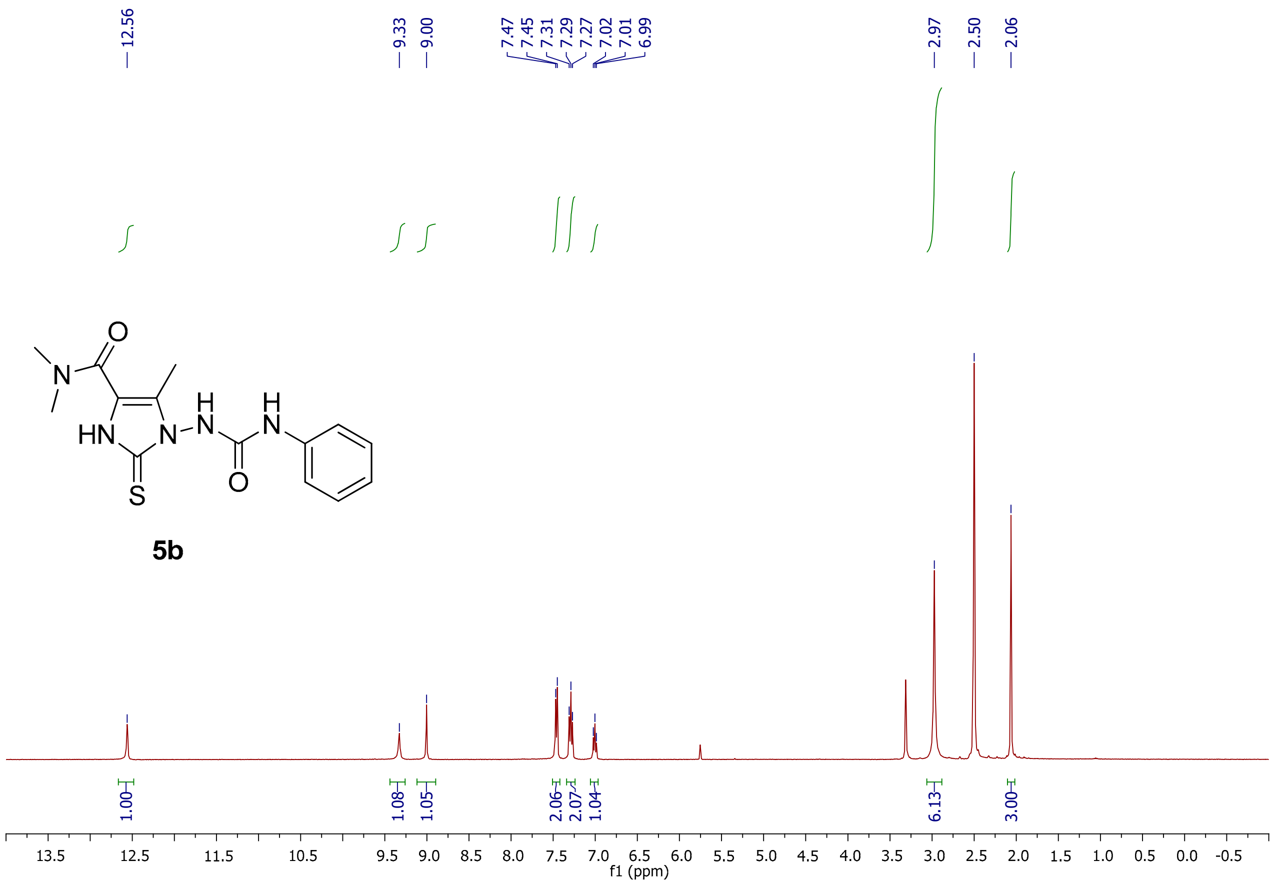
12.56
9.33
9.00
7.47
7.45
7.31
7.29
7.27
7.02
7.01
6.99
2.97
2.50
2.06
5b
1.00
1.08
1.05
2.06
2.07
1.04
6.13
3.00
f1 (ppm)

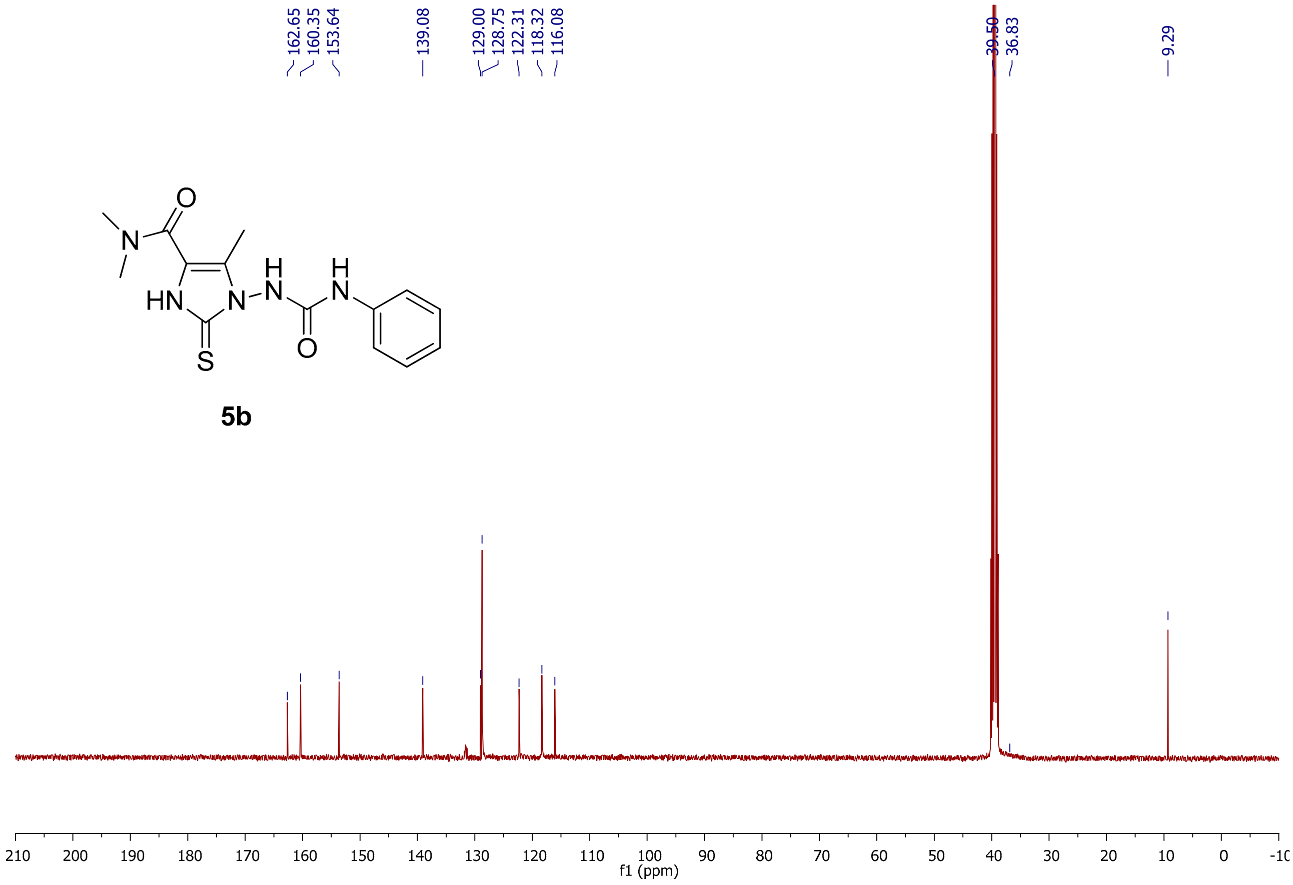
162.65
160.35
153.64
139.08
129.00
128.75
122.31
118.32
116.08
39.50
36.83
9.29
5b
f1 (ppm)

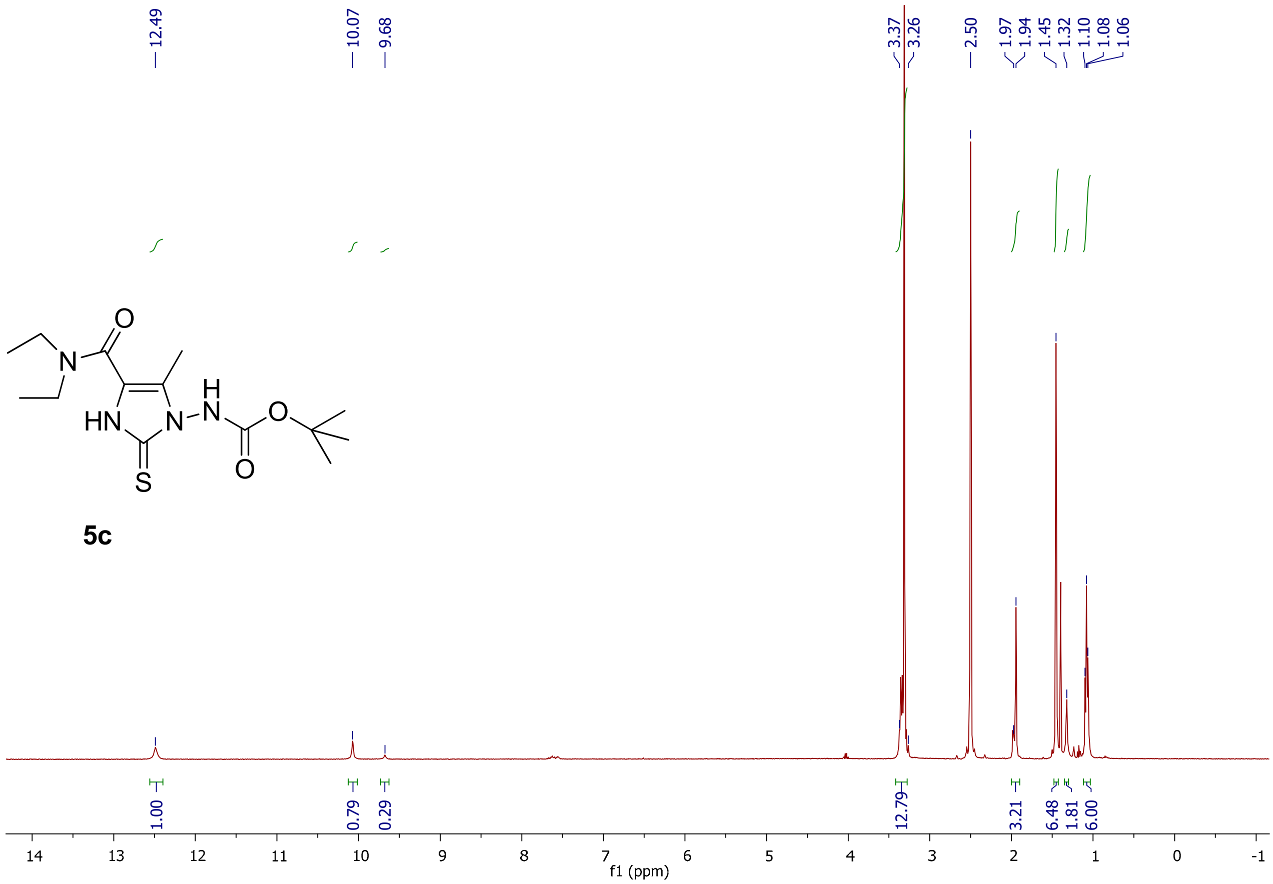
12.49
10.07
9.68
3.37
3.26
2.50
1.97
1.94
1.45
1.32
1.10
1.08
1.06
O
N
H
HN
N
N
O
S
O
5c
1.00
0.79
0.29
12.79
3.21
6.48
1.81
6.00
14 13 12 11 10 9 8 7 6 5 4 3 2 1 0 -1
f1 (ppm)

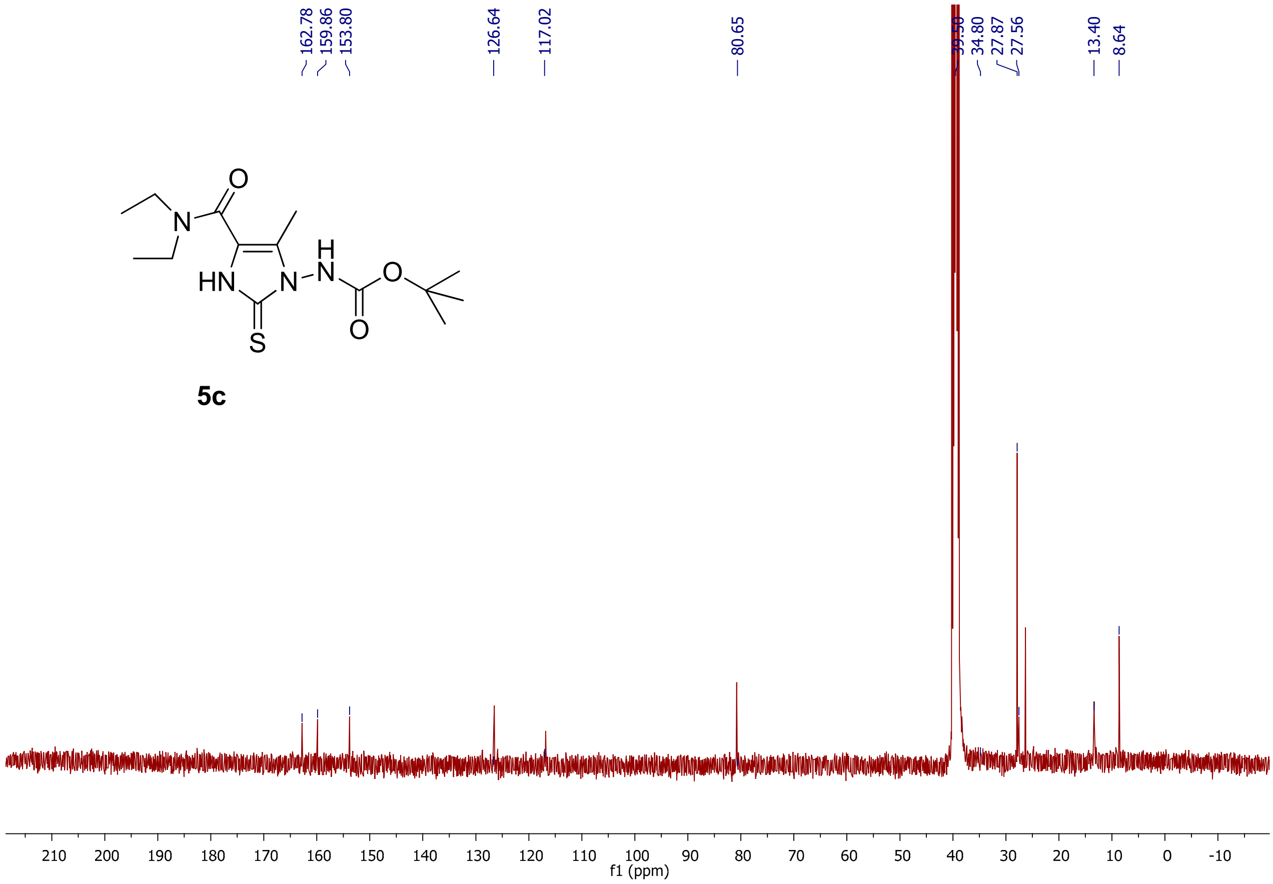
162.78
159.86
153.80
126.64
117.02
80.65
34.80
27.87
27.56
13.40
8.64
O
N
H
HN
N
N
O
S
O
5c
210 200 190 180 170 160 150 140 130 120 110 100 90 80 70 60 50 40 30 20 10 0 -10
f1 (ppm)

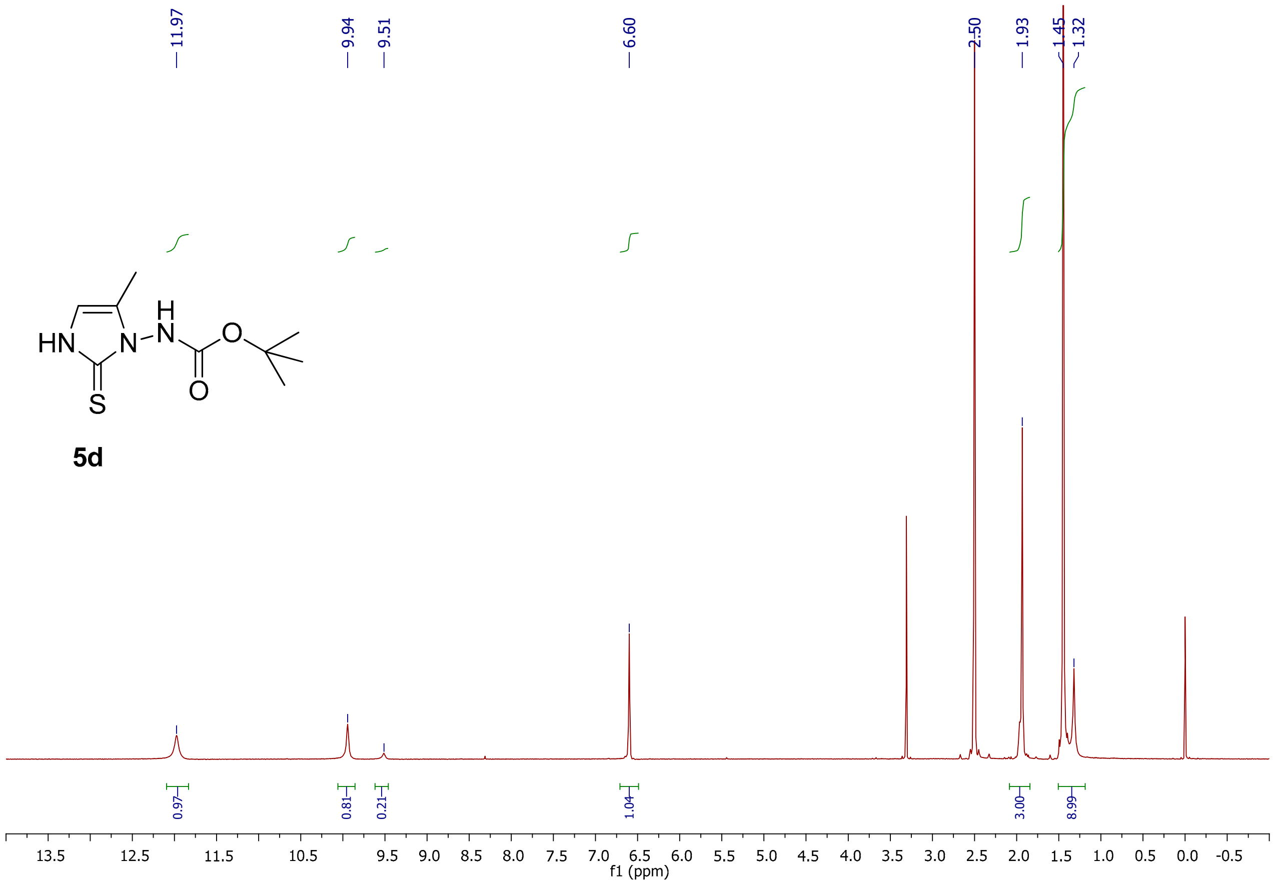
11.97
9.94
9.51
6.60
2.50
1.93
1.45
1.32
5d
0.97
0.81
0.21
1.04
3.00
8.99
f1 (ppm)

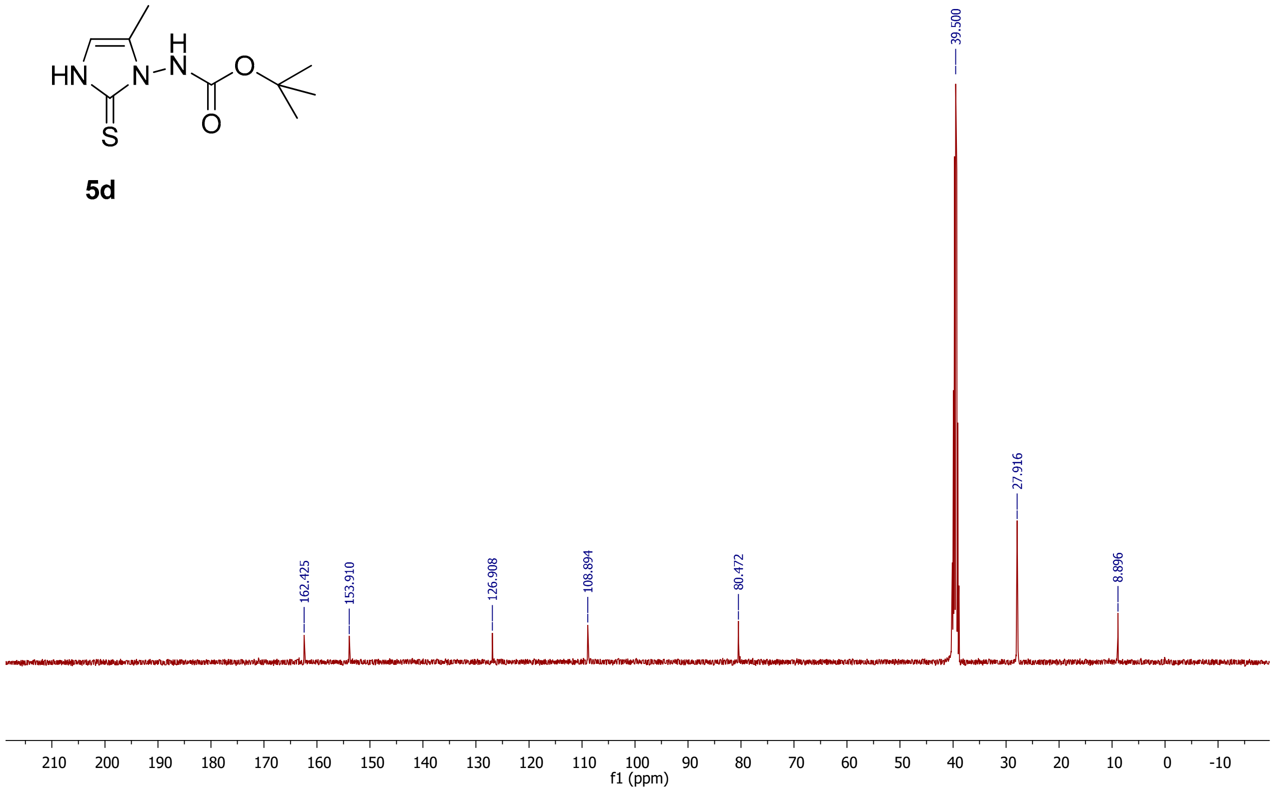
39.500
27.916
162.425
153.910
126.908
108.894
80.472
8.896
5d
f1 (ppm)

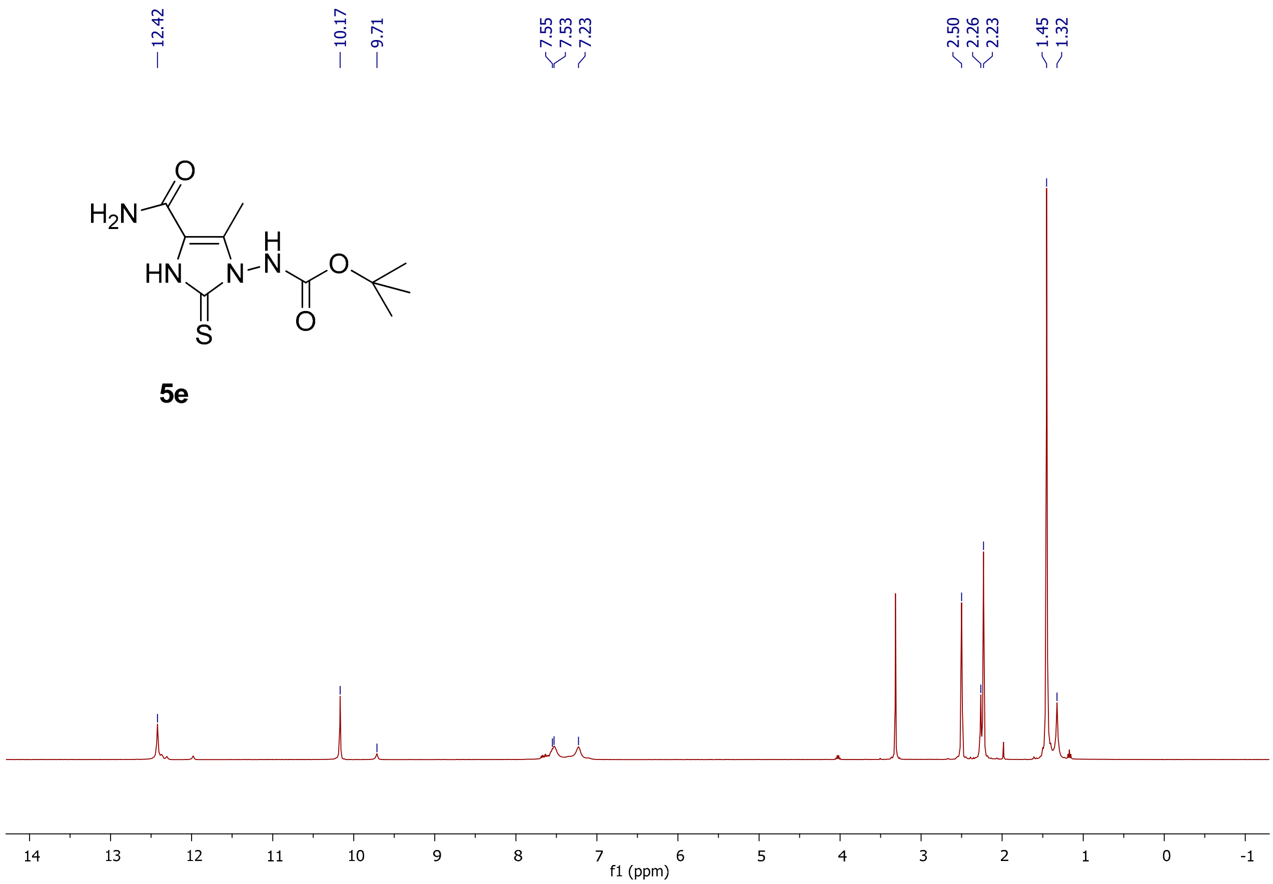
12.42
10.17
9.71
7.55
7.53
7.23
2.50
2.26
2.23
1.45
1.32
5e
f1 (ppm)

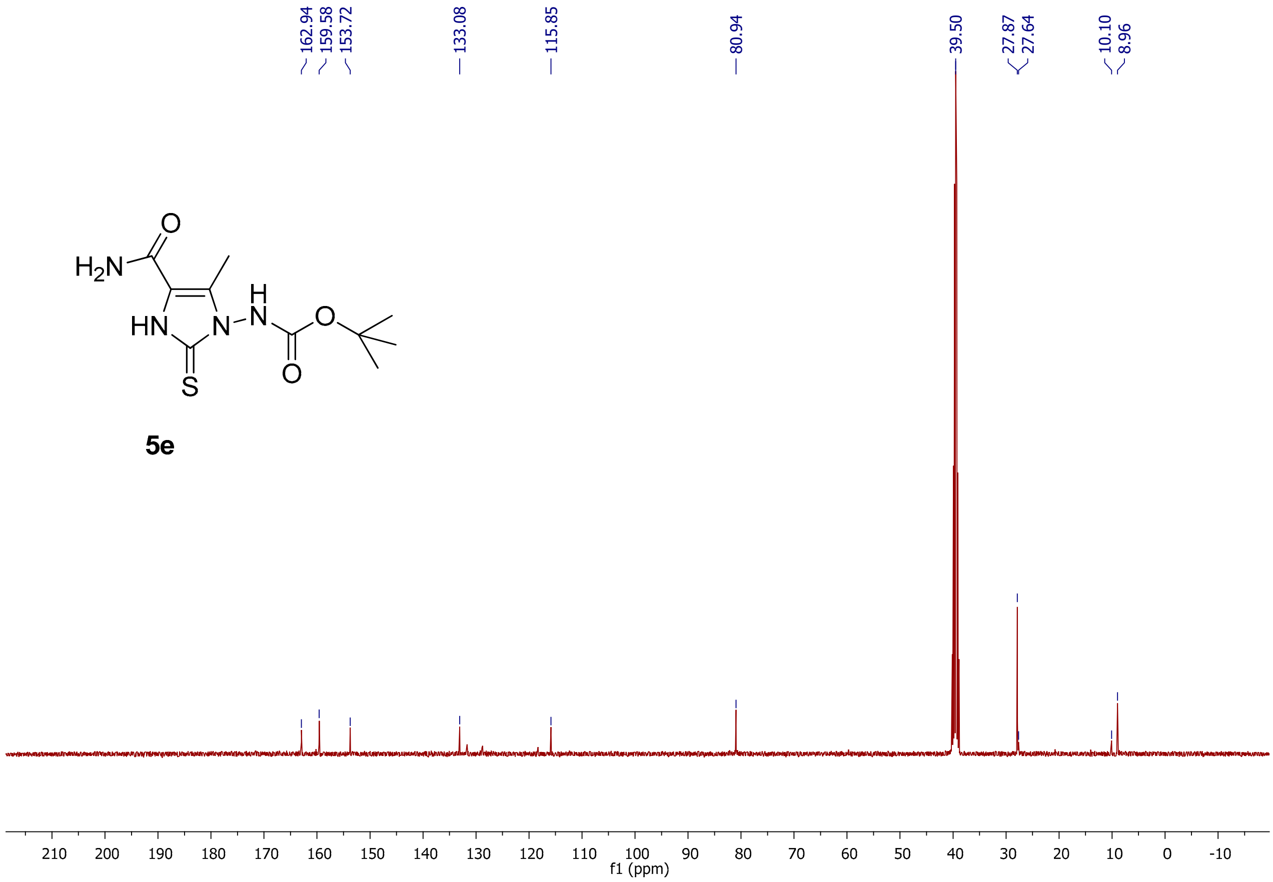
162.94
159.58
153.72
133.08
115.85
80.94
39.50
27.87
27.64
10.10
8.96
5e
f1 (ppm)

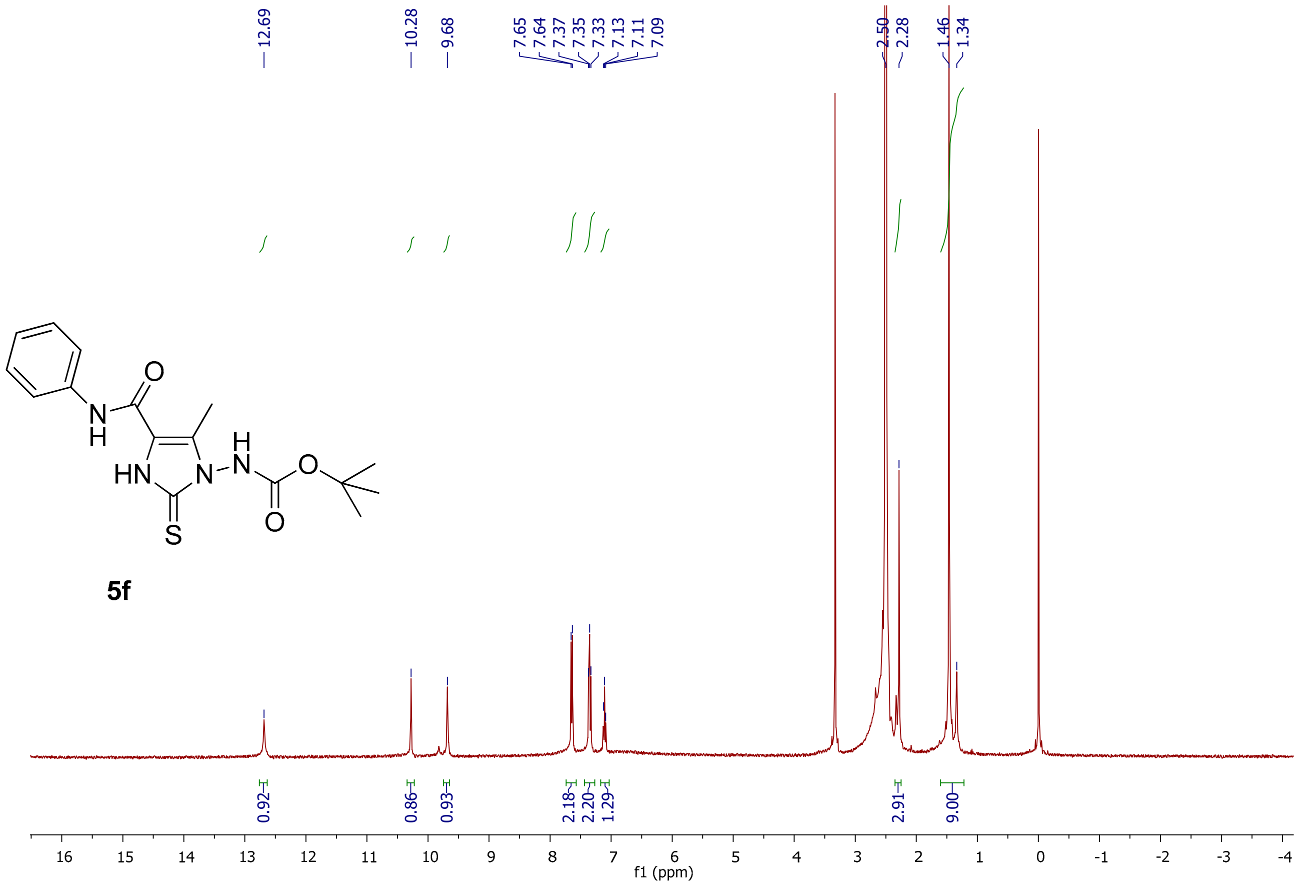
12.69
10.28
9.68
7.65
7.64
7.37
7.35
7.33
7.13
7.11
7.09
2.50
2.28
1.46
1.34
5f
0.92
0.86
0.93
2.18
2.20
1.29
2.91
9.00
f1 (ppm)

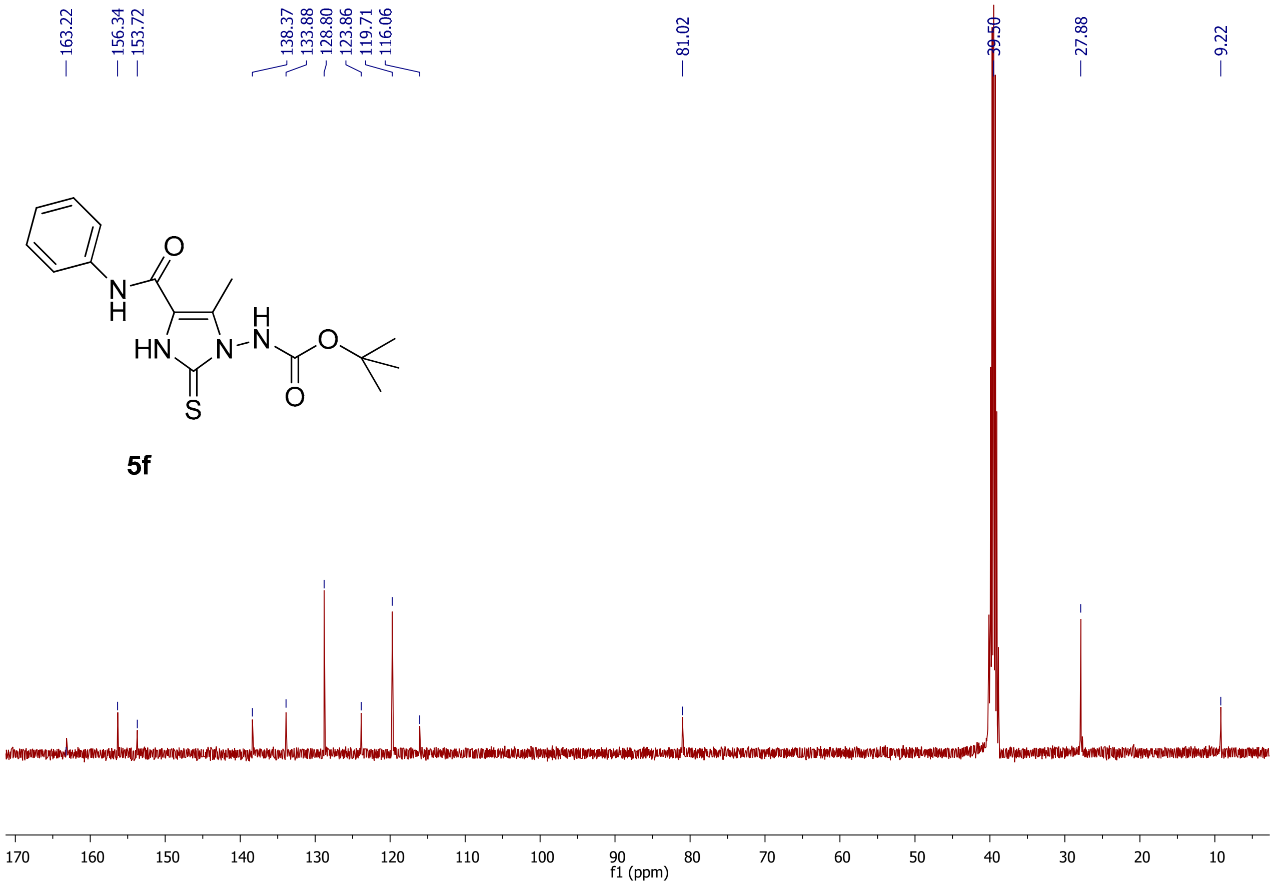
163.22
156.34
153.72
138.37
133.88
128.80
123.86
119.71
116.06
81.02
39.50
27.88
9.22
5f
f1 (ppm)

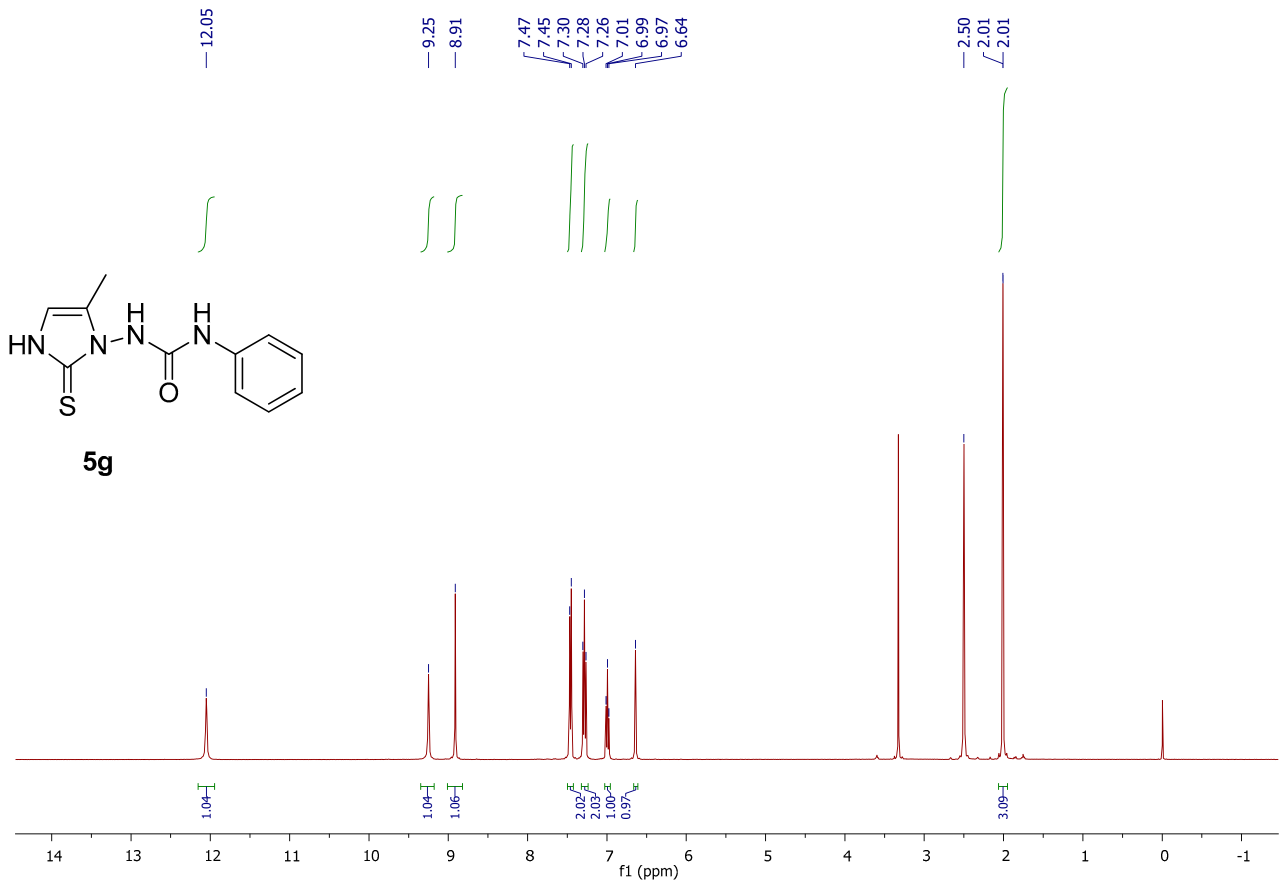
12.05
9.25
8.91
7.47
7.45
7.30
7.28
7.26
7.01
6.99
6.97
6.64
2.50
2.01
2.01
HN
N
H
N
H
N
S
O
5g
1.04
1.04
1.06
2.02
2.03
1.00
0.97
3.09
14 13 12 11 10 9 8 7 6 5 4 3 2 1 0 -1
f1 (ppm)

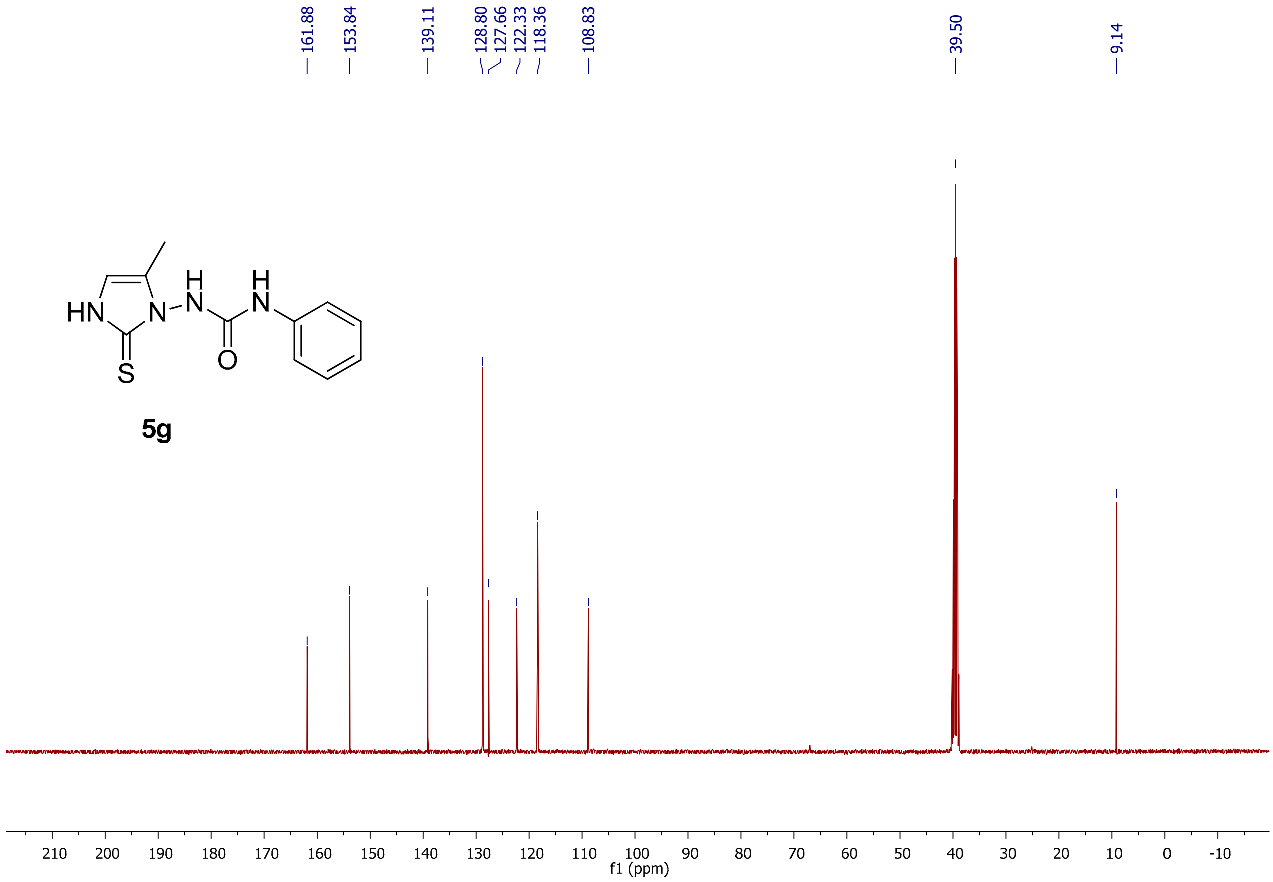
161.88
153.84
139.11
128.80
127.66
122.33
118.36
108.83
39.50
9.14
HN
N
H
N
H
N
S
O
5g
210 200 190 180 170 160 150 140 130 120 110 100 90 80 70 60 50 40 30 20 10 0 -10
f1 (ppm)

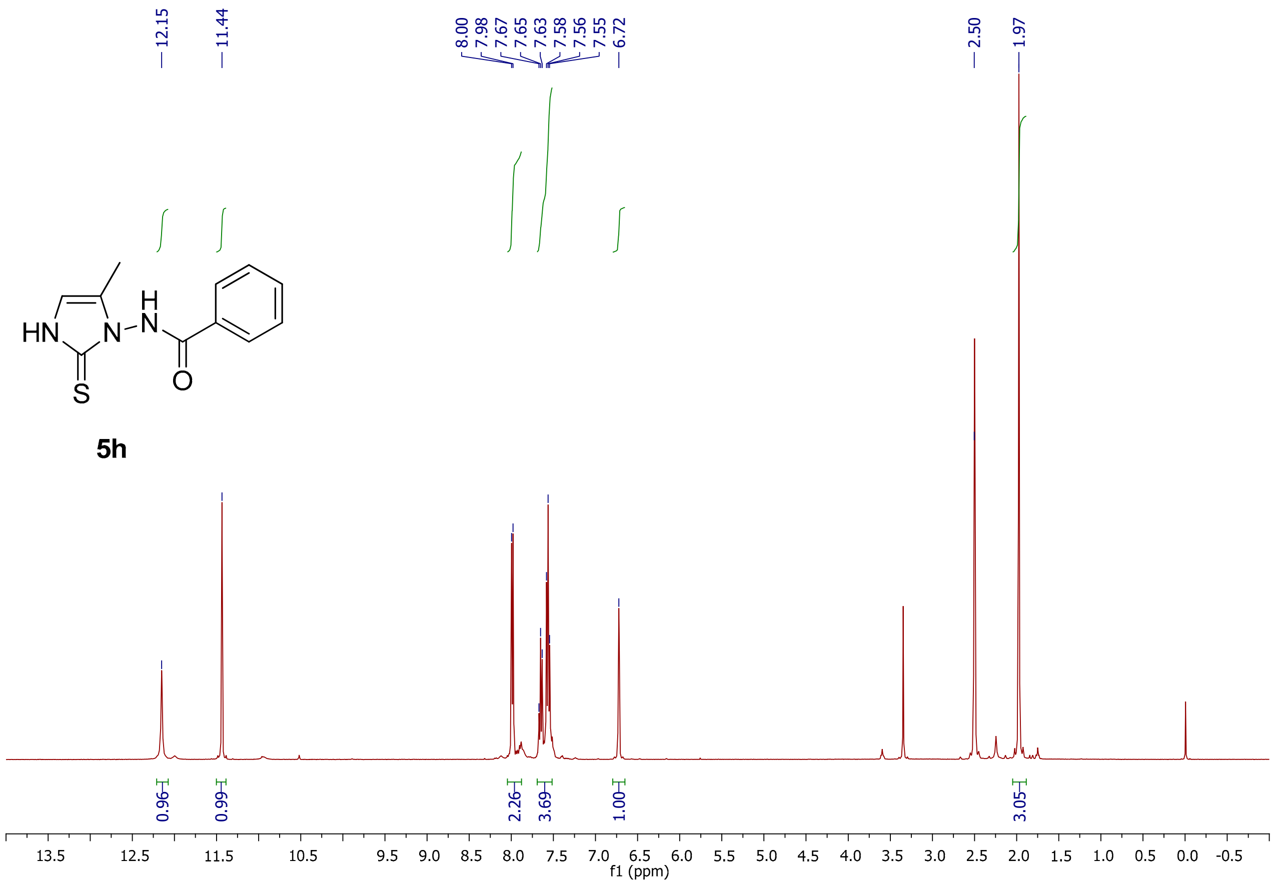
12.15
11.44
8.00
7.98
7.67
7.65
7.63
7.58
7.56
7.55
6.72
2.50
1.97
HN
N
H
N
S
O
5h
0.96
0.99
2.26
3.69
1.00
3.05
13.5 12.5 11.5 10.5 9.5 9.0 8.5 8.0 7.5 7.0 6.5 6.0 5.5 5.0 4.5 4.0 3.5 3.0 2.5 2.0 1.5 1.0 0.5 0.0 -0.5
f1 (ppm)

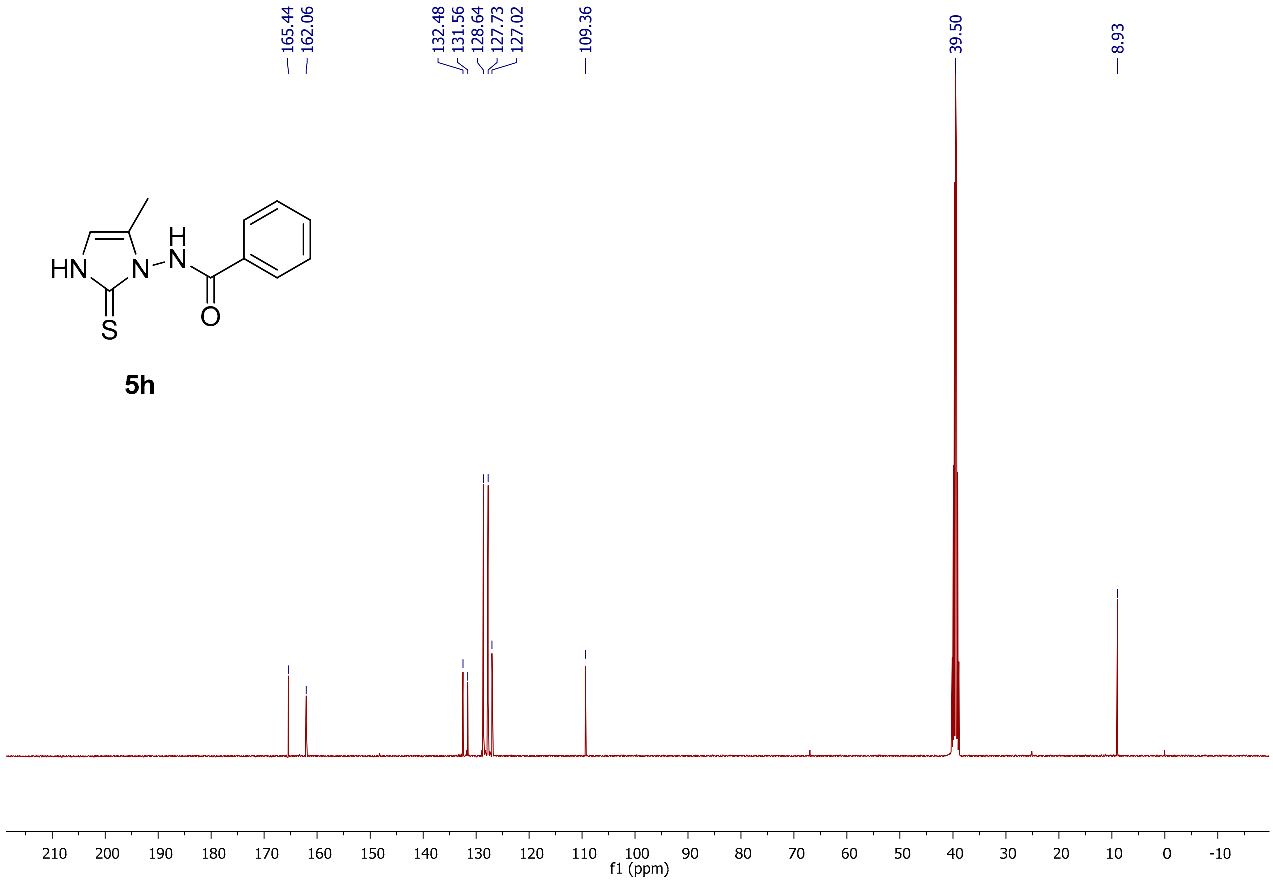
165.44
162.06
132.48
131.56
128.64
127.73
127.02
109.36
39.50
8.93
HN
N
H
N
S
O
5h
210 200 190 180 170 160 150 140 130 120 110 100 90 80 70 60 50 40 30 20 10 0 -10
f1 (ppm)

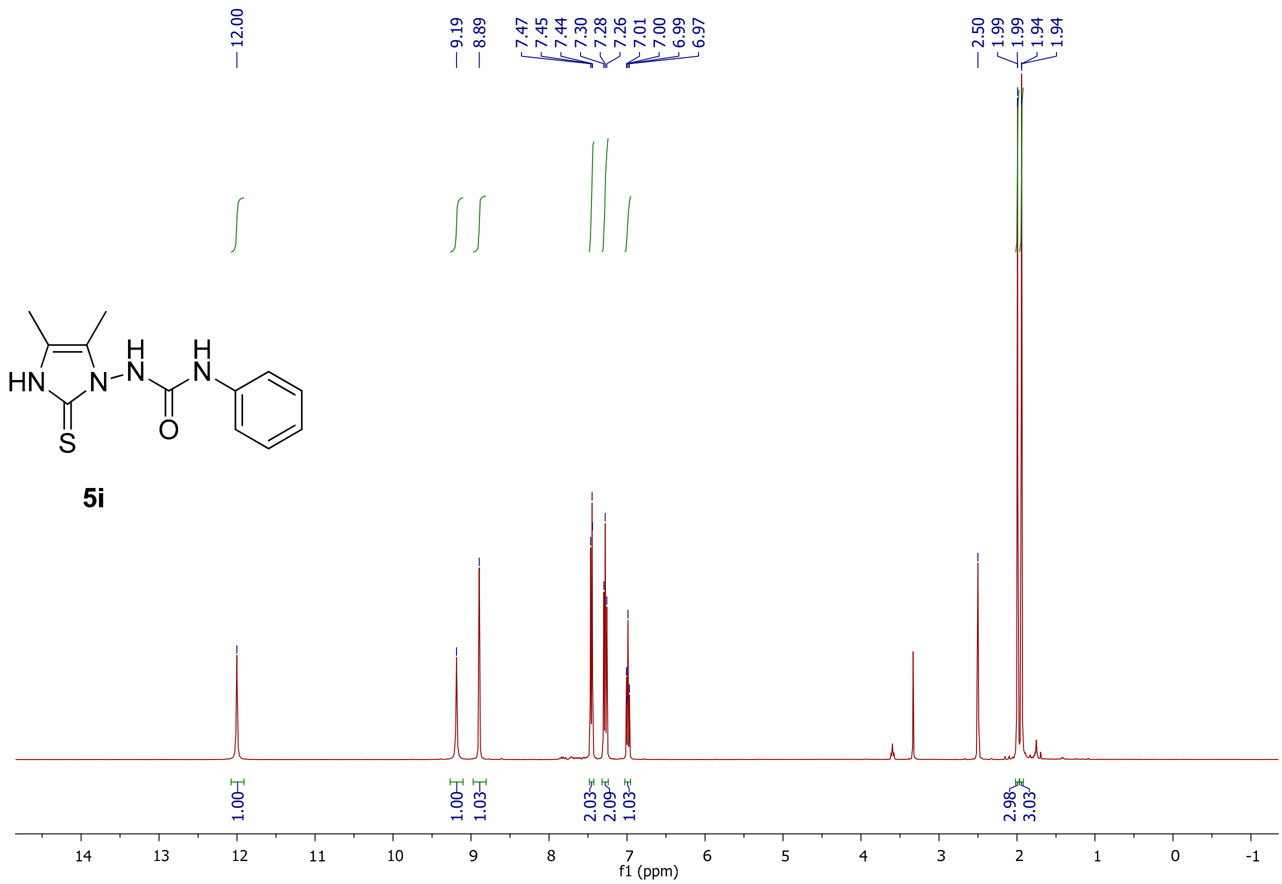

12.00
9.19
8.89
7.47
7.45
7.44
7.30
7.28
7.26
7.01
7.00
6.99
6.97
2.50
1.99
1.99
1.94
1.94
HN
N
H
N
H
N
O
S
5i
1.00
1.00
1.03
2.03
2.09
1.03
2.98
3.03
14 13 12 11 10 9 8 7 6 5 4 3 2 1 0 -1
f1 (ppm)

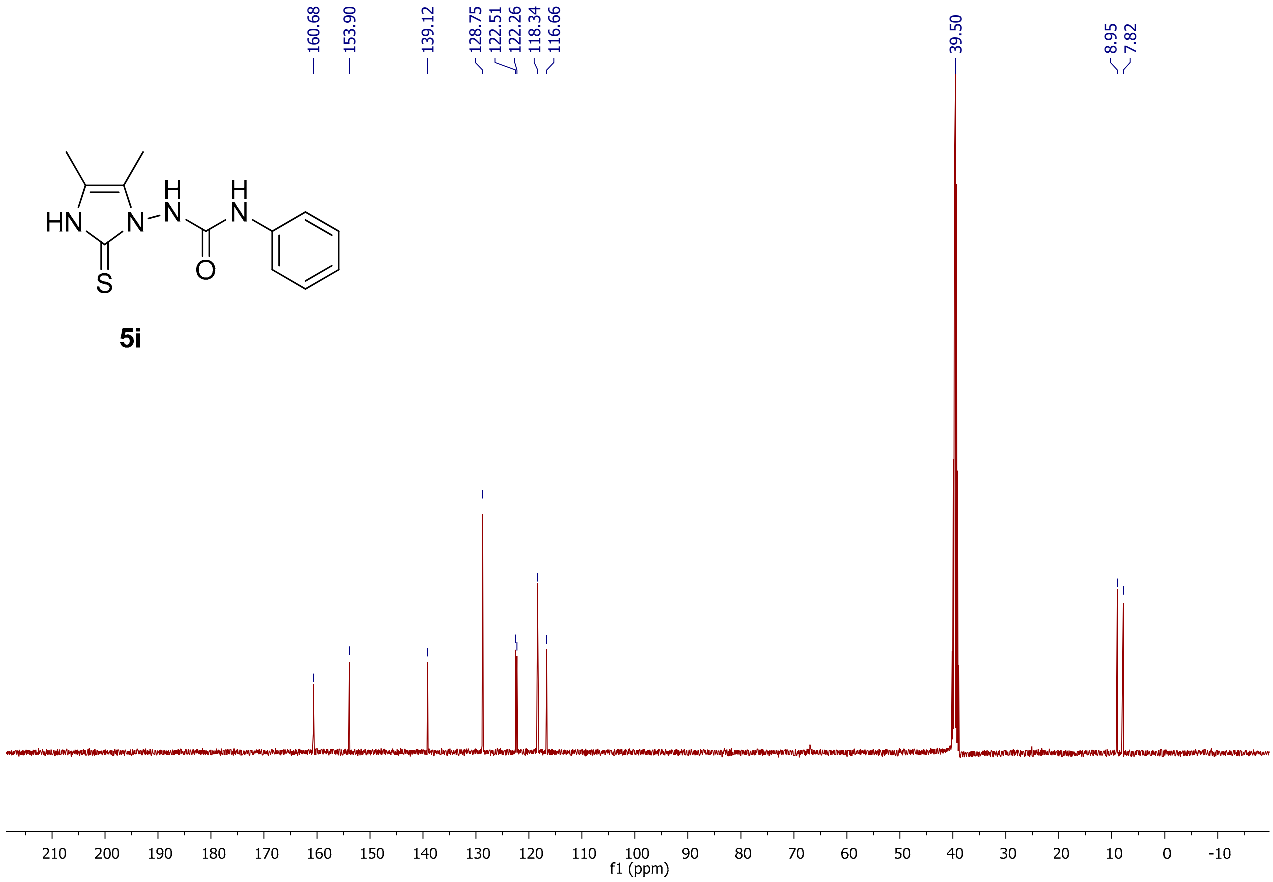

160.68
153.90
139.12
128.75
122.51
122.26
118.34
116.66
39.50
8.95
7.82
HN
N
H
N
H
N
O
S
5i
210 200 190 180 170 160 150 140 130 120 110 100 90 80 70 60 50 40 30 20 10 0 -10
f1 (ppm)

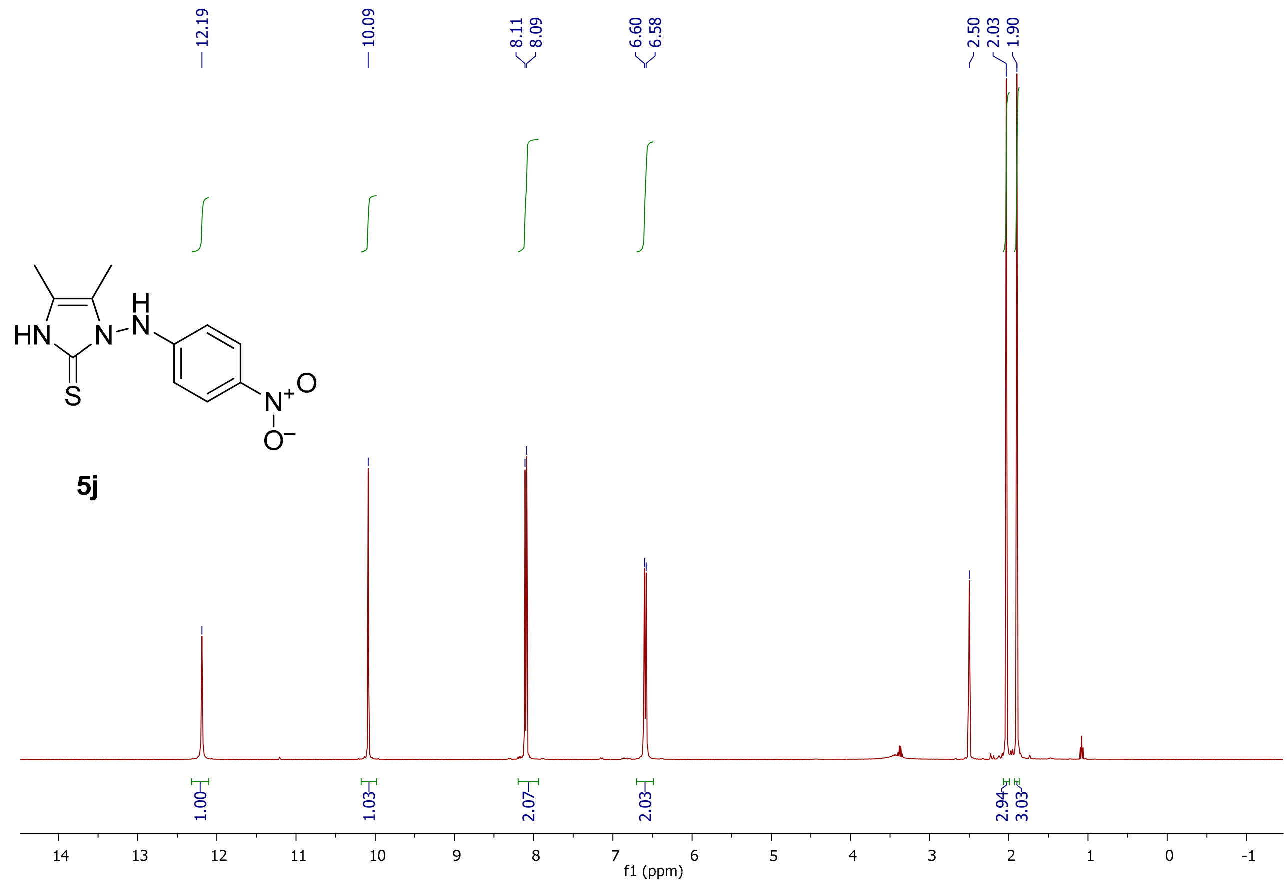
12.19
10.09
8.11
8.09
6.60
6.58
2.50
2.03
1.90
HN
N
H
N
S
N+
O
O−
5j
1.00
1.03
2.07
2.03
2.94
3.03
f1 (ppm)

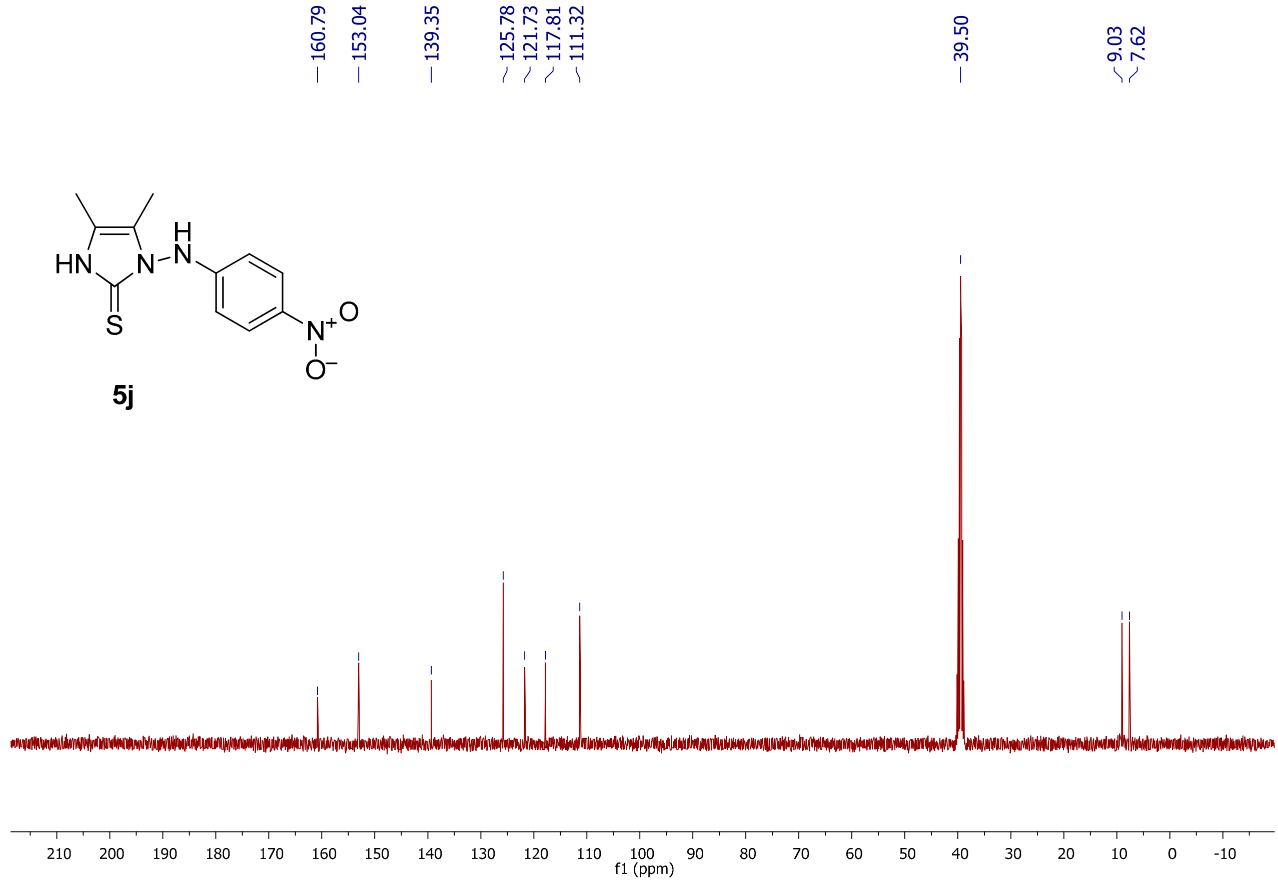
160.79
153.04
139.35
125.78
121.73
117.81
111.32
39.50
9.03
7.62
HN
N
H
N
S
N+
O
O−
5j
f1 (ppm)

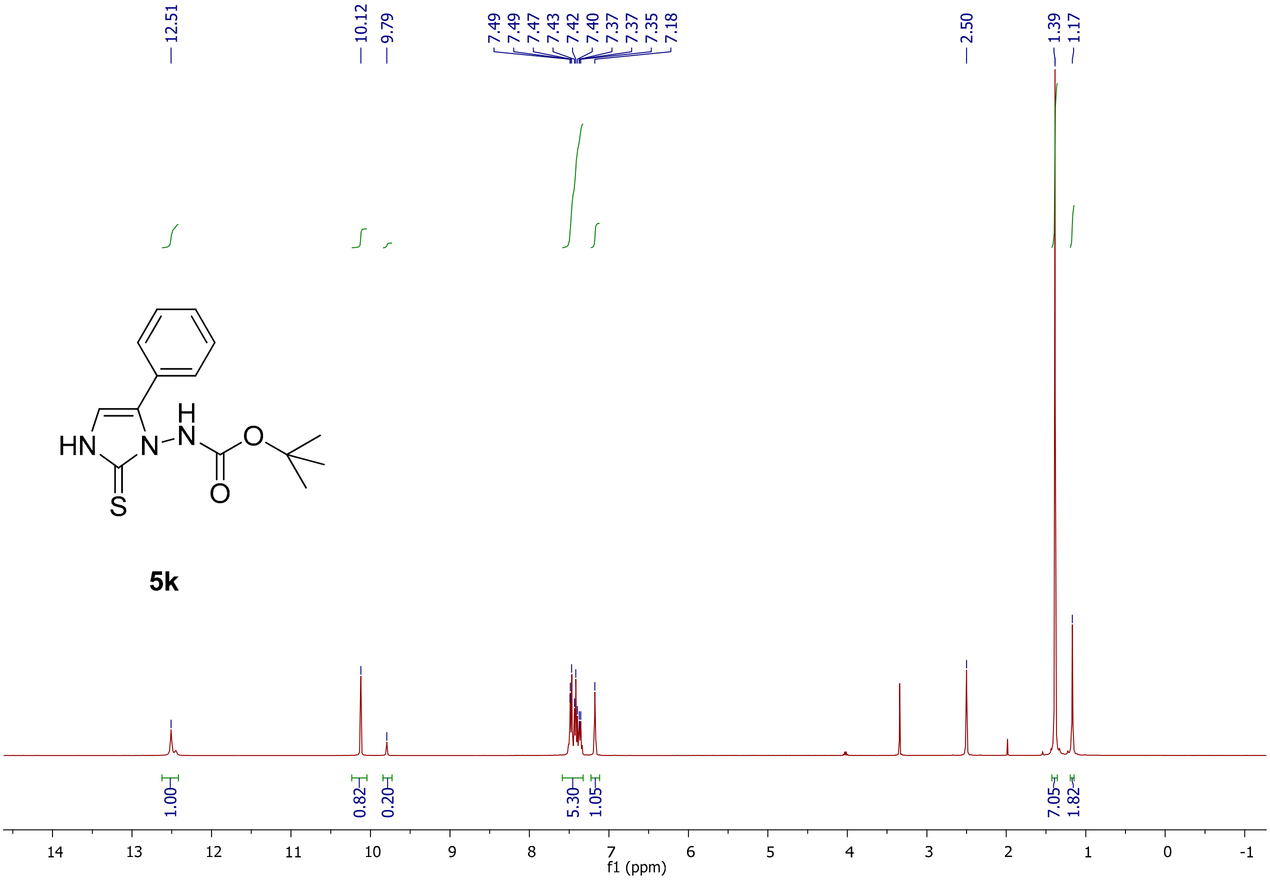
12.51
10.12
9.79
7.49 7.49 7.47 7.43 7.42 7.40 7.37 7.37 7.35 7.18
2.50
1.39
1.17
HN
N
H
N
O
O
S
5k
1.00
0.82
0.20
5.30
1.05
7.05
1.82
14 13 12 11 10 9 8 7 6 5 4 3 2 1 0 -1
f1 (ppm)

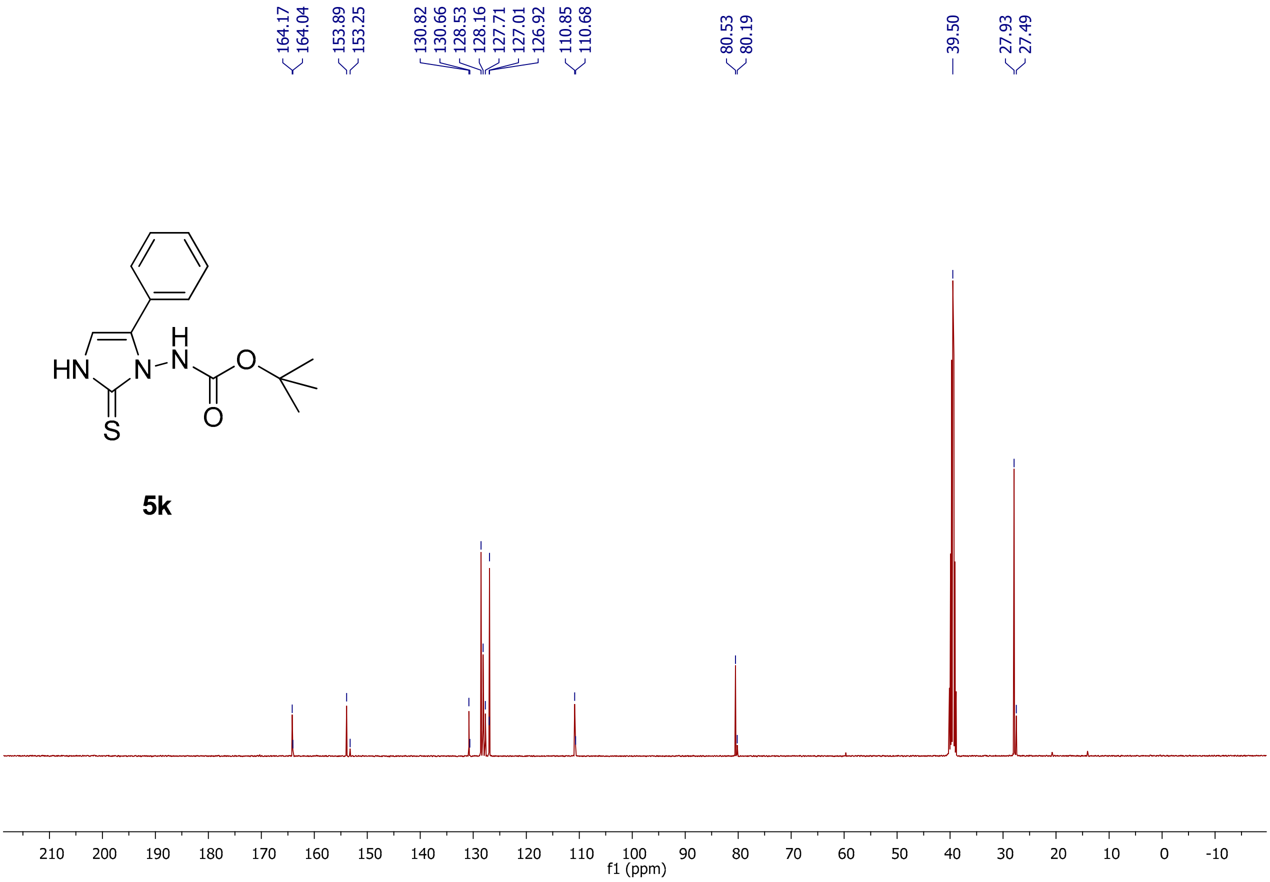
164.17
164.04
153.89
153.25
130.82 130.66 128.53 128.16 127.71 127.01 126.92
110.85
110.68
80.53
80.19
39.50
27.93
27.49
HN
N
H
N
O
O
S
5k
210 200 190 180 170 160 150 140 130 120 110 100 90 80 70 60 50 40 30 20 10 0 -10
f1 (ppm)

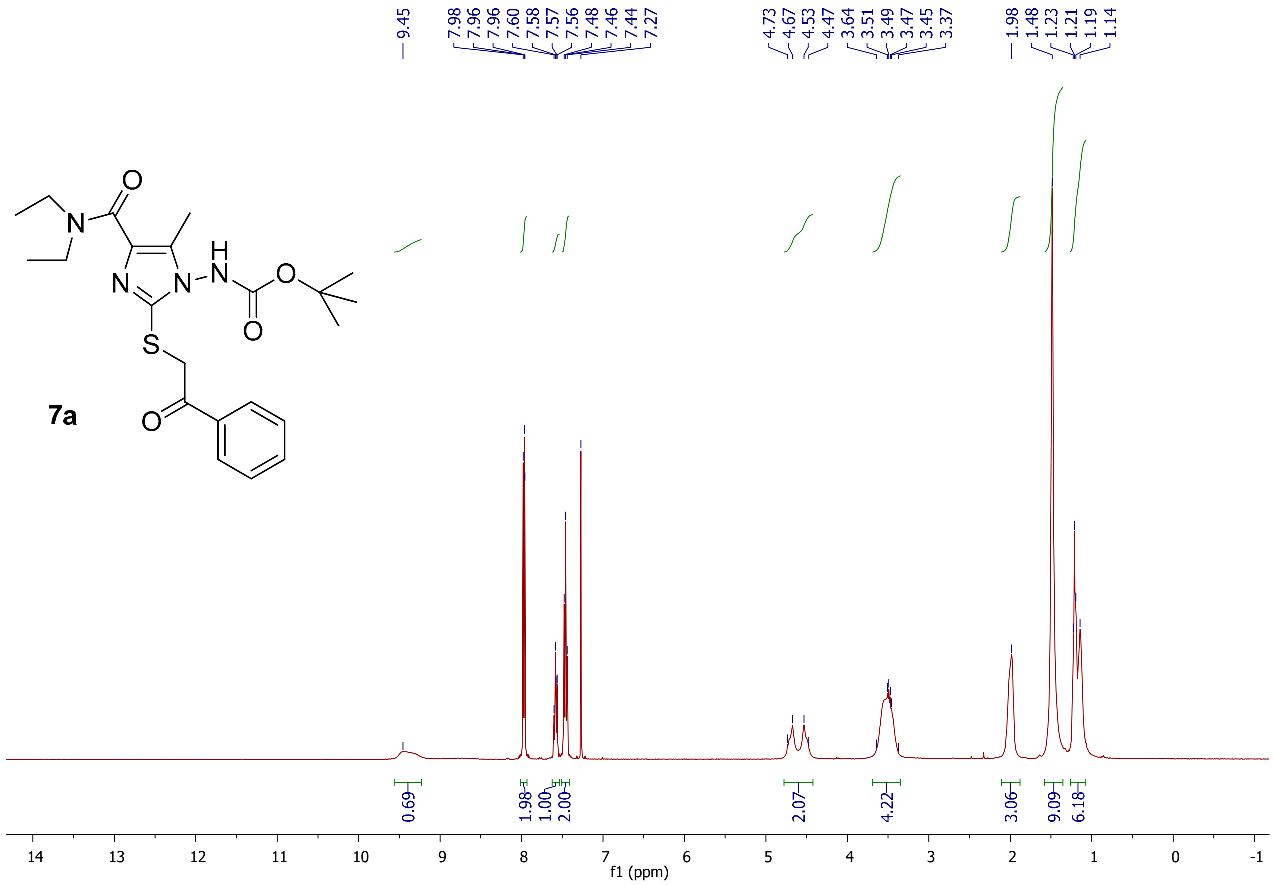
9.45
7.98
7.96
7.96
7.60
7.58
7.57
7.56
7.48
7.46
7.44
7.27
4.73
4.67
4.53
4.47
3.64
3.51
3.49
3.47
3.45
3.37
1.98
1.48
1.23
1.21
1.19
1.14
7a
0.69
1.98
1.00
2.00
2.07
4.22
3.06
9.09
6.18
14 13 12 11 10 9 8 7 6 5 4 3 2 1 0 -1
f1 (ppm)

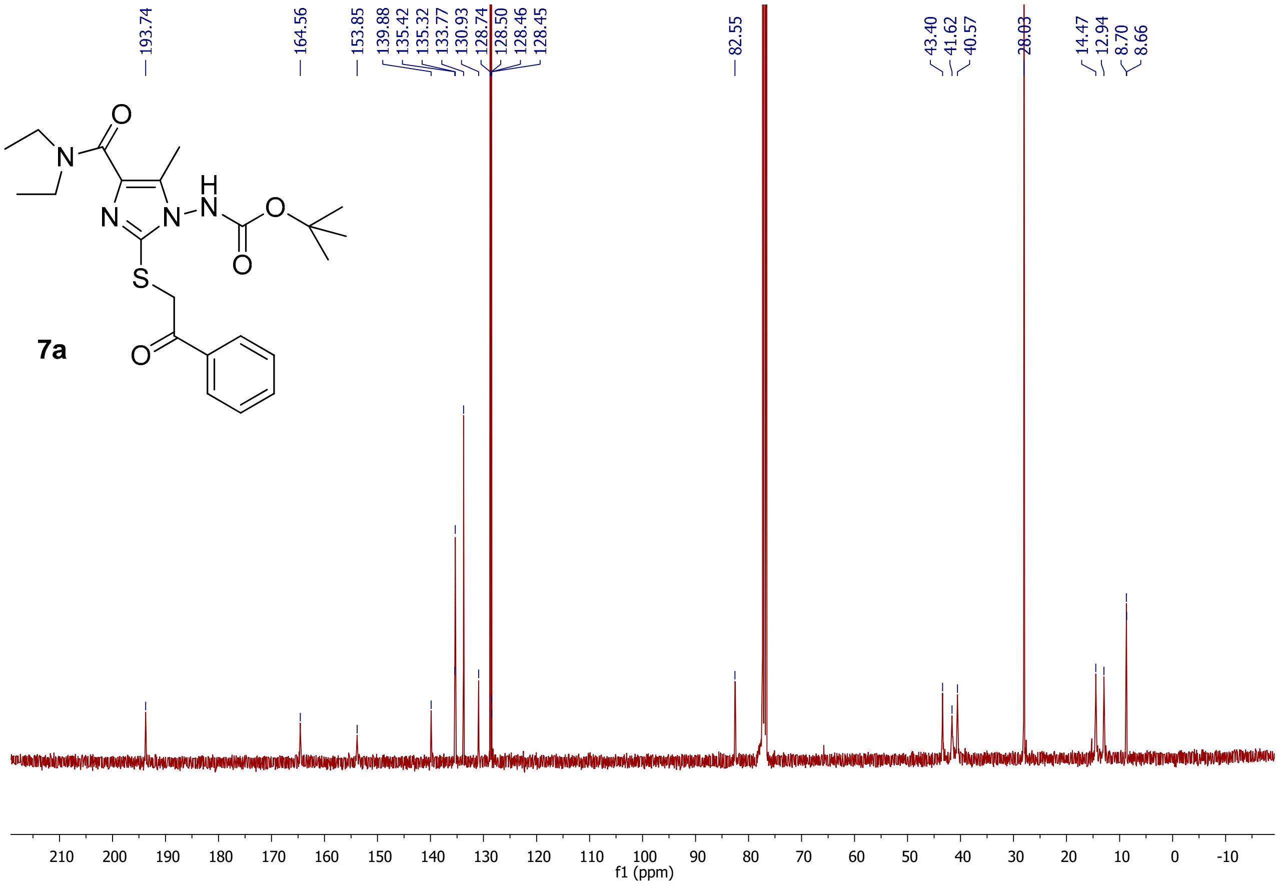
193.74
164.56
153.85
139.88
135.42
135.32
133.77
130.93
128.74
128.50
128.46
128.45
82.55
43.40
41.62
40.57
28.03
14.47
12.94
8.70
8.66
7a
210 200 190 180 170 160 150 140 130 120 110 100 90 80 70 60 50 40 30 20 10 0 -10
f1 (ppm)

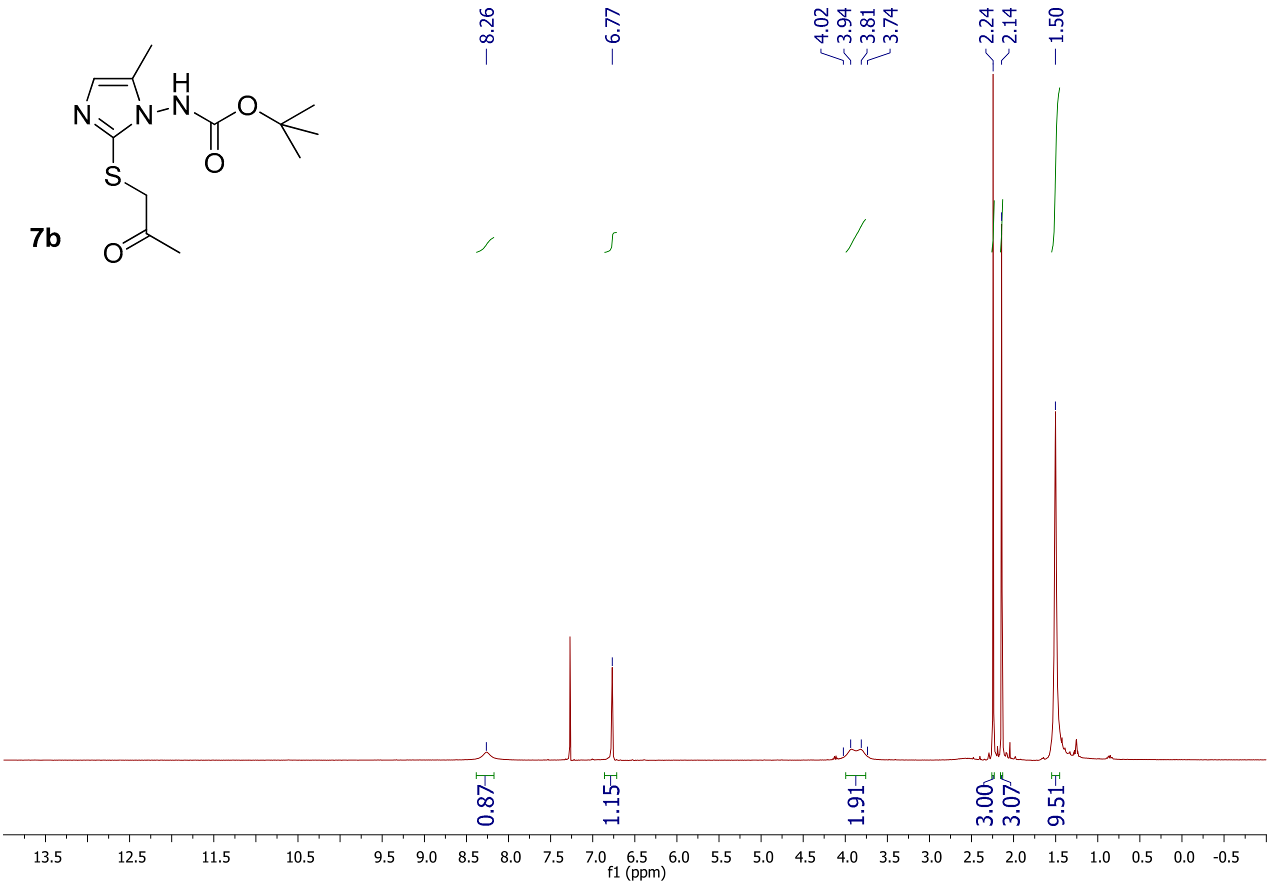
7b
8.26
6.77
4.02
3.94
3.81
3.74
2.24
2.14
1.50
0.87
1.15
1.91
3.00
3.07
9.51
f1 (ppm)

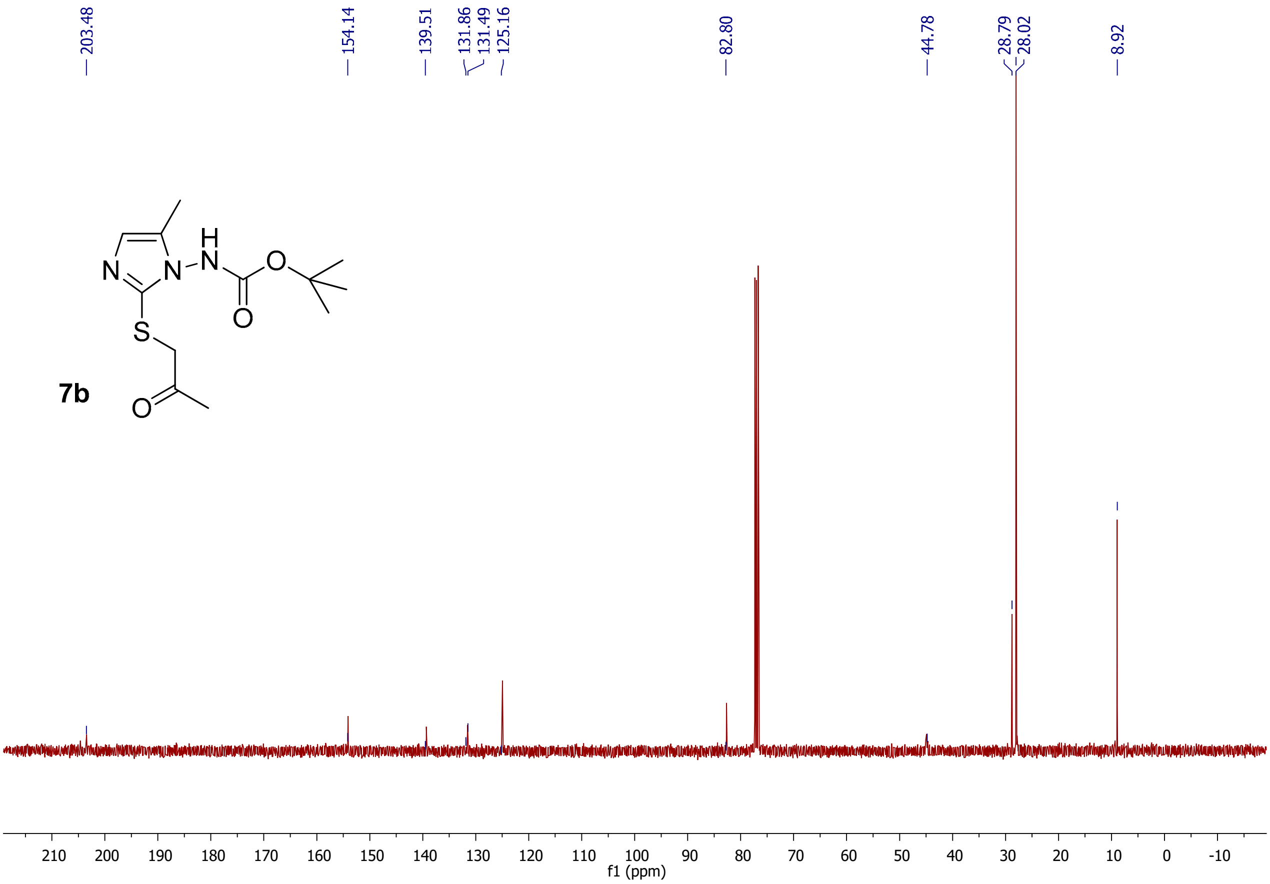
7b
203.48
154.14
139.51
131.86
131.49
125.16
82.80
44.78
28.79
28.02
8.92
f1 (ppm)

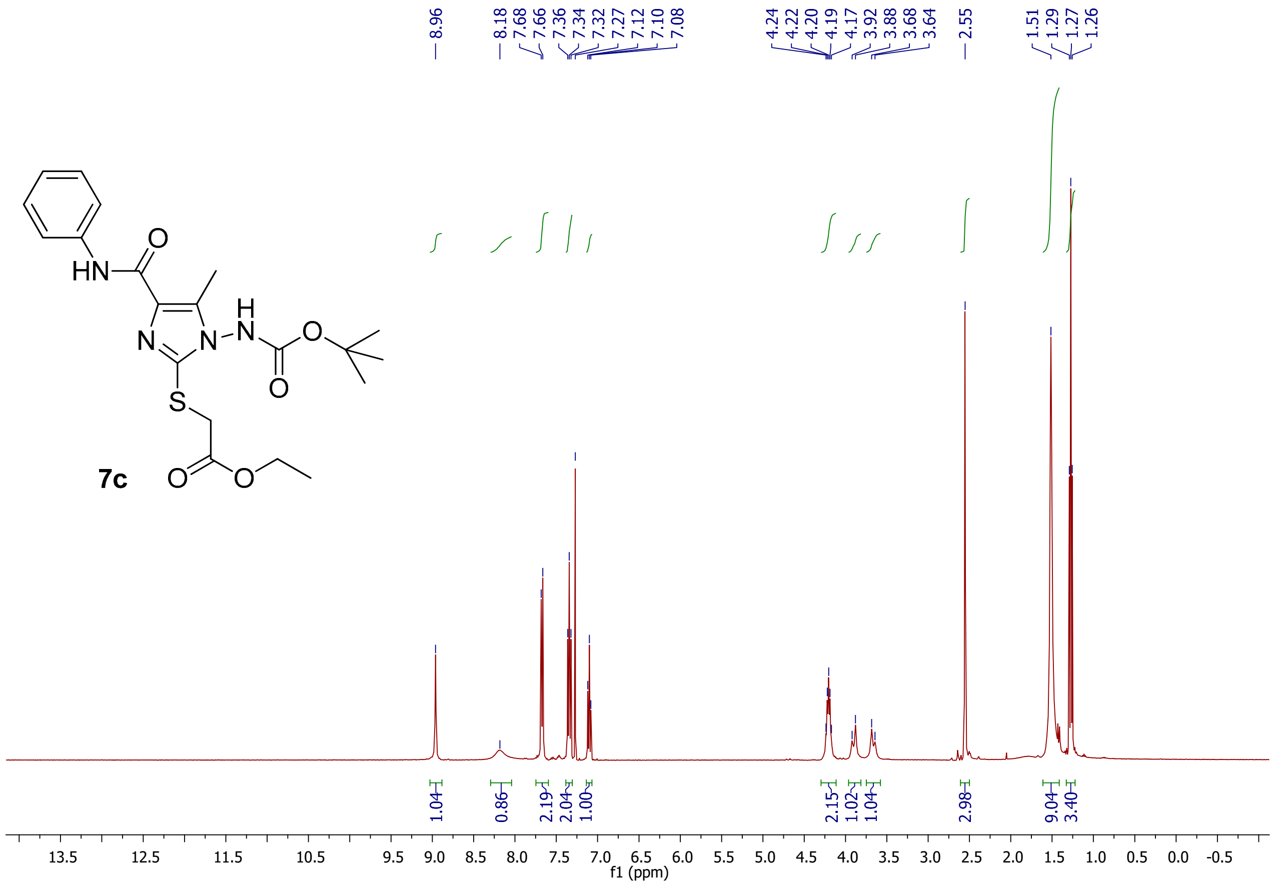
8.96
8.18
7.68
7.66
7.36
7.34
7.32
7.27
7.12
7.10
7.08
4.24
4.22
4.20
4.19
4.17
3.92
3.88
3.68
3.64
2.55
1.51
1.29
1.27
1.26
7c
1.04
0.86
2.19
2.04
1.00
2.15
1.02
1.04
2.98
9.04
3.40
13.5 12.5 11.5 10.5 9.5 9.0 8.5 8.0 7.5 7.0 6.5 6.0 5.5 5.0 4.5 4.0 3.5 3.0 2.5 2.0 1.5 1.0 0.5 0.0 -0.5
f1 (ppm)

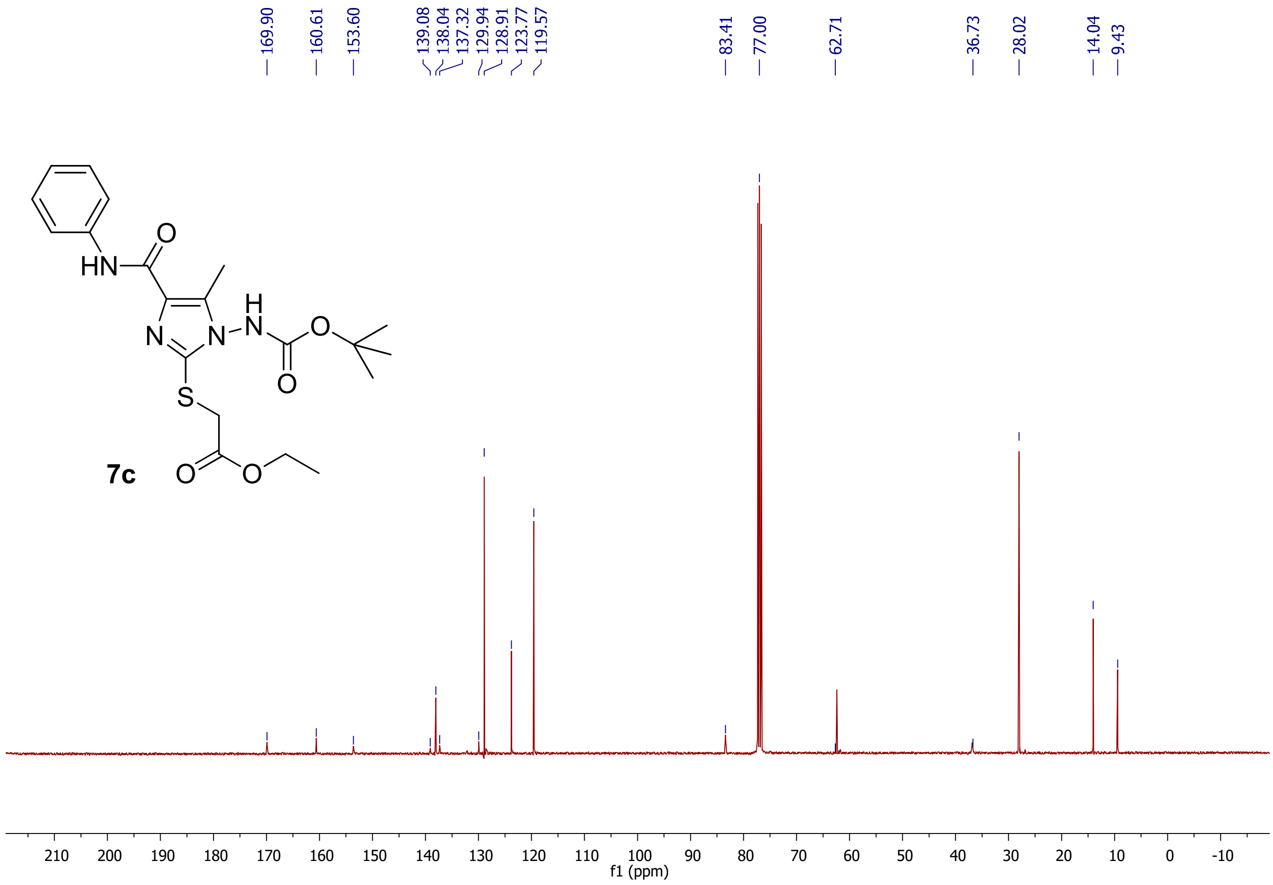
169.90
160.61
153.60
139.08
138.04
137.32
129.94
128.91
123.77
119.57
83.41
77.00
62.71
36.73
28.02
14.04
9.43
7c
210 200 190 180 170 160 150 140 130 120 110 100 90 80 70 60 50 40 30 20 10 0 -10
f1 (ppm)

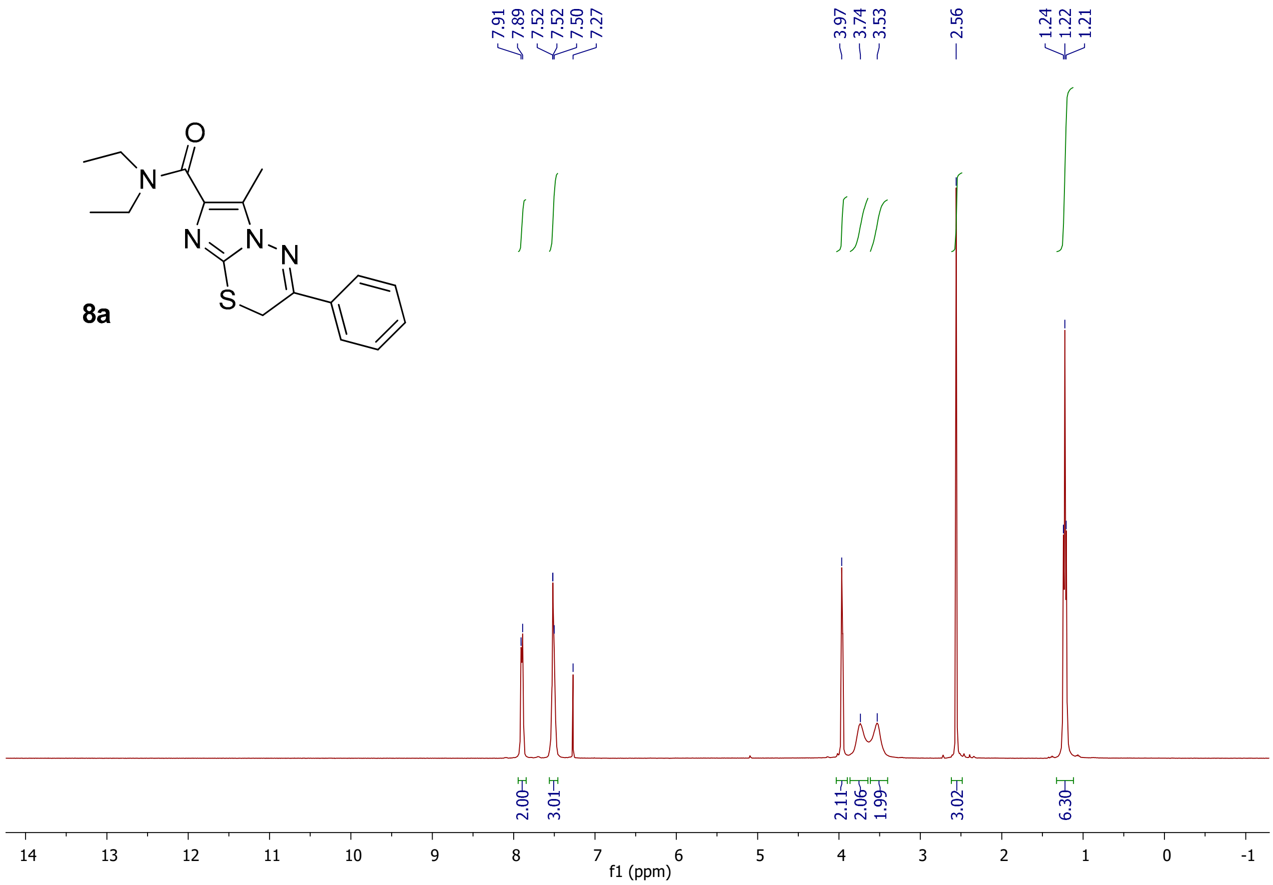
8a
7.91
7.89
7.52
7.52
7.50
7.27
3.97
3.74
3.53
2.56
1.24
1.22
1.21
2.00
3.01
2.11
2.06
1.99
3.02
6.30
f1 (ppm)

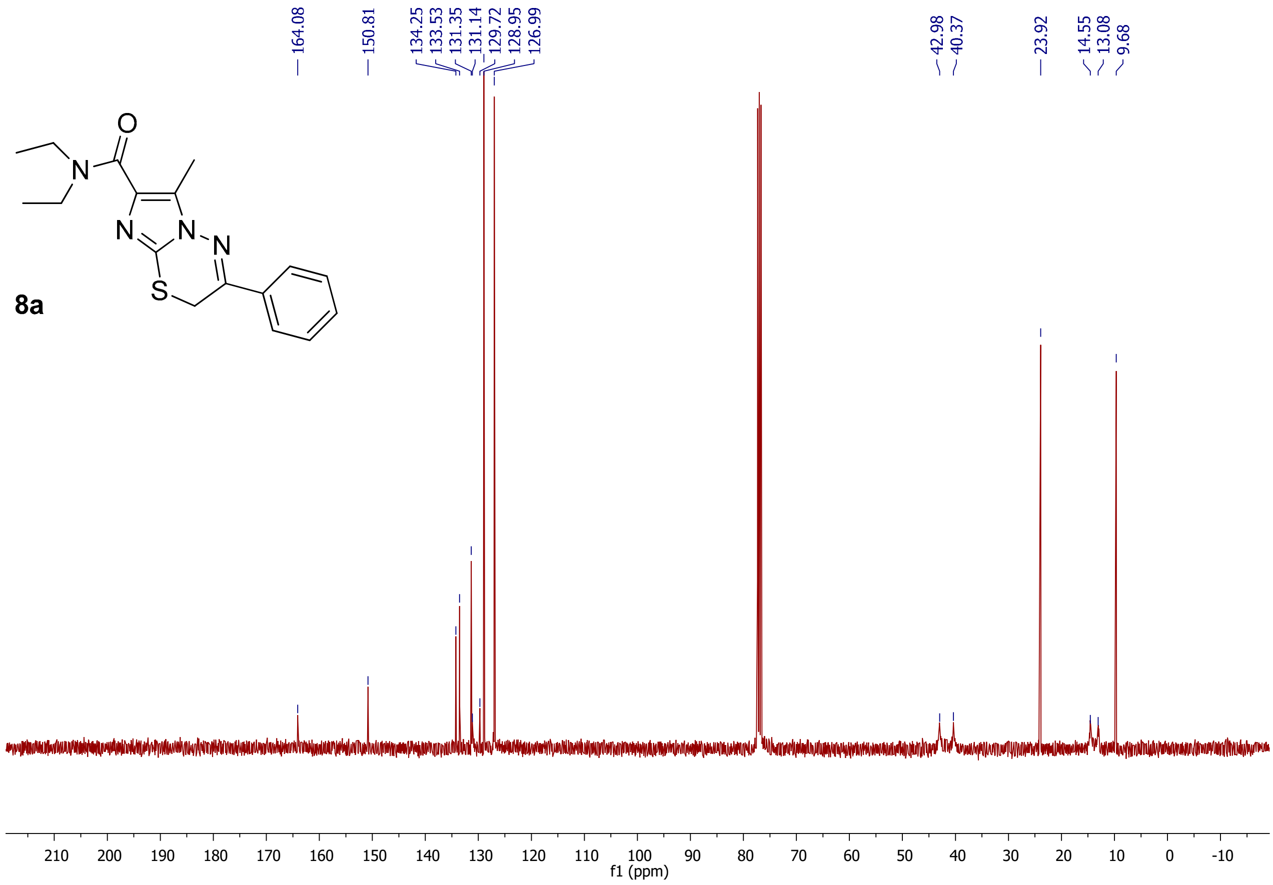
8a
164.08
150.81
134.25
133.53
131.35
131.14
129.72
128.95
126.99
42.98
40.37
23.92
14.55
13.08
9.68
f1 (ppm)

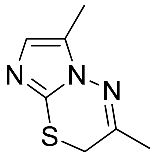

8b

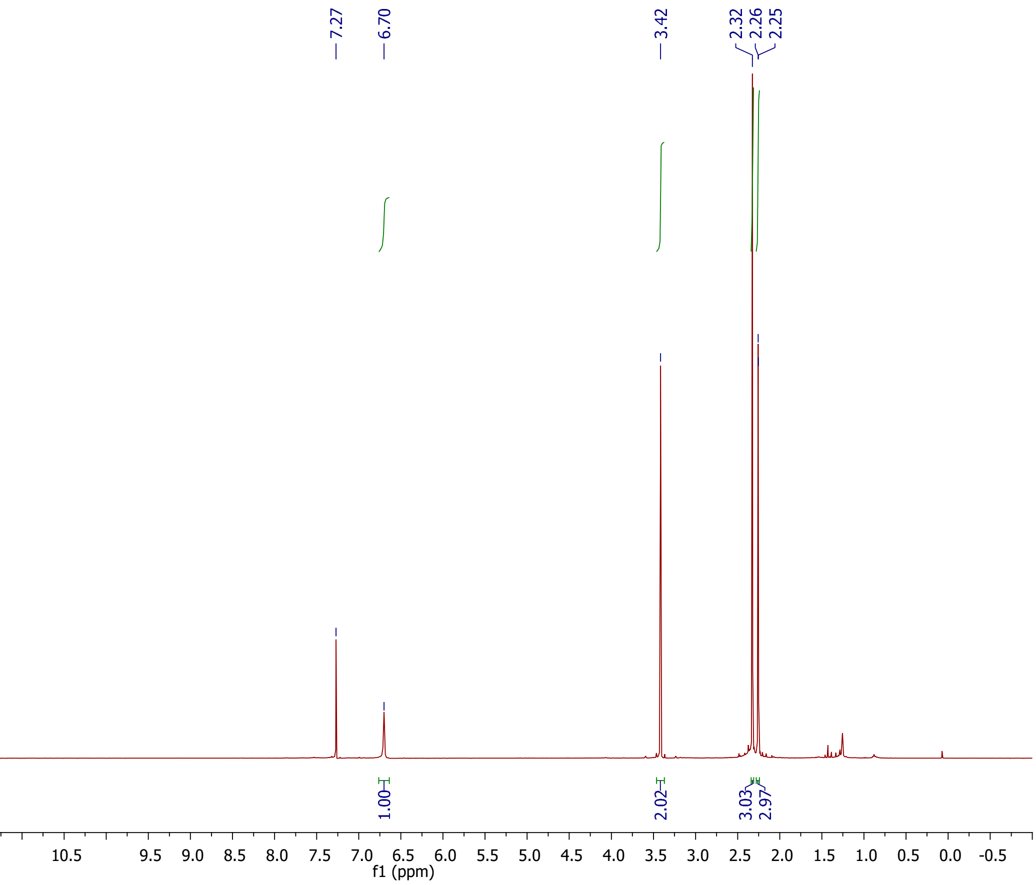

7.27
6.70
3.42
2.32
2.26
2.25
1.00
2.02
3.03
2.97
13.5 12.5 11.5 10.5 9.5 9.0 8.5 8.0 7.5 7.0 6.5 6.0 5.5 5.0 4.5 4.0 3.5 3.0 2.5 2.0 1.5 1.0 0.5 0.0 -0.5
f1 (ppm)

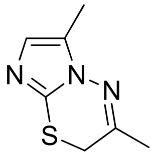

8b

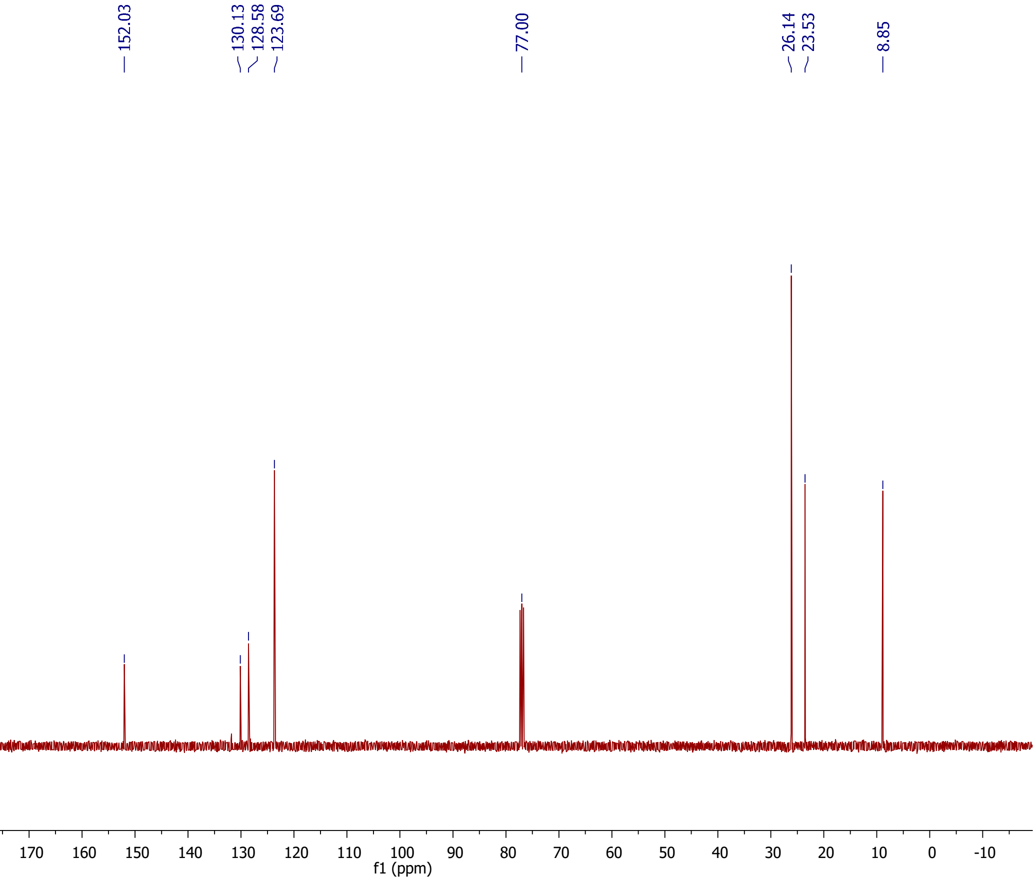

152.03
130.13
128.58
123.69
77.00
26.14
23.53
8.85
210 200 190 180 170 160 150 140 130 120 110 100 90 80 70 60 50 40 30 20 10 0 -10
f1 (ppm)

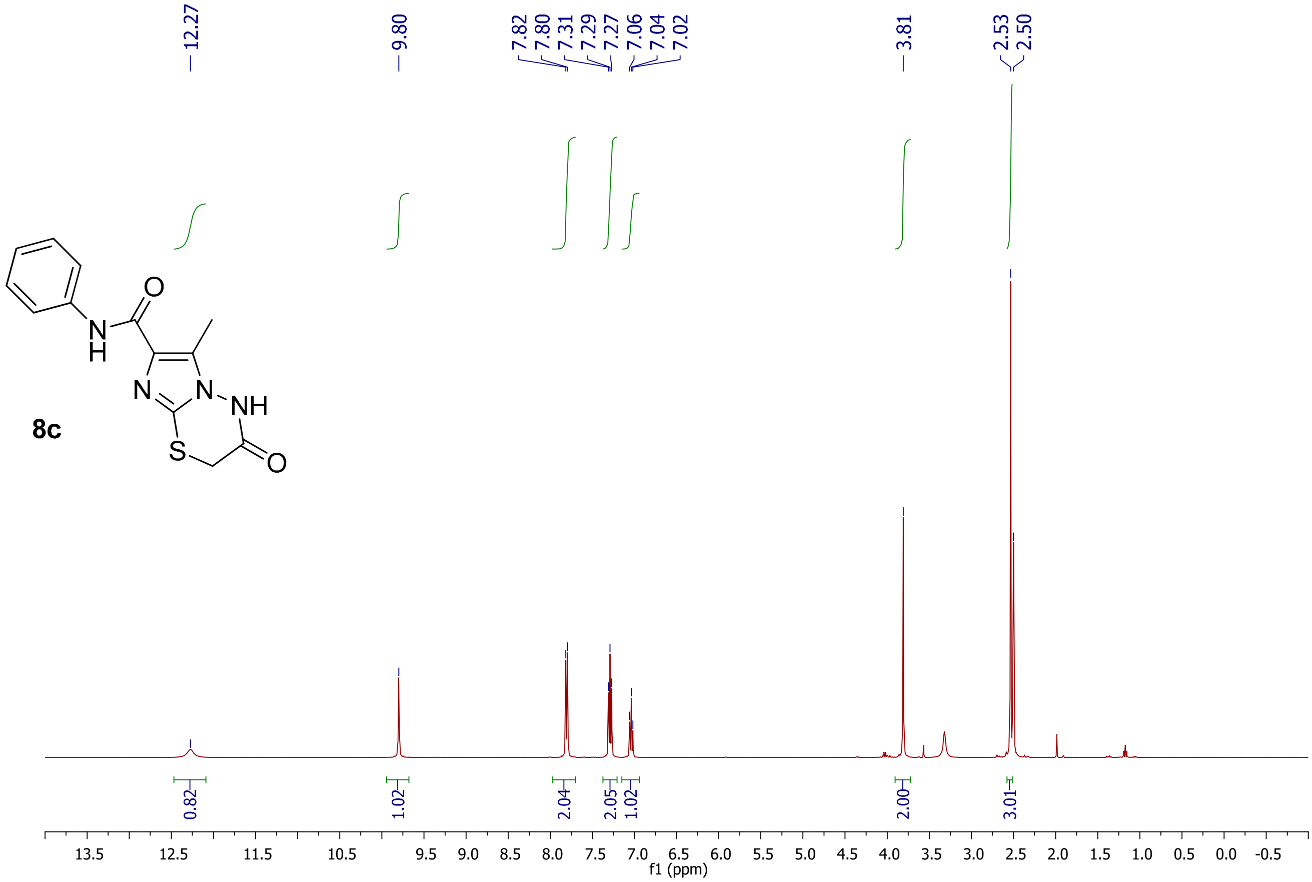
12.27
9.80
7.82
7.80
7.31
7.29
7.27
7.06
7.04
7.02
3.81
2.53
2.50
8c
O
N
H
N
N
NH
S
O
0.82
1.02
2.04
2.05
1.02
2.00
3.01
13.5
12.5
11.5
10.5
9.5
9.0
8.5
8.0
7.5
7.0
6.5
6.0
5.5
5.0
4.5
4.0
3.5
3.0
2.5
2.0
1.5
1.0
0.5
0.0
-0.5
f1 (ppm)

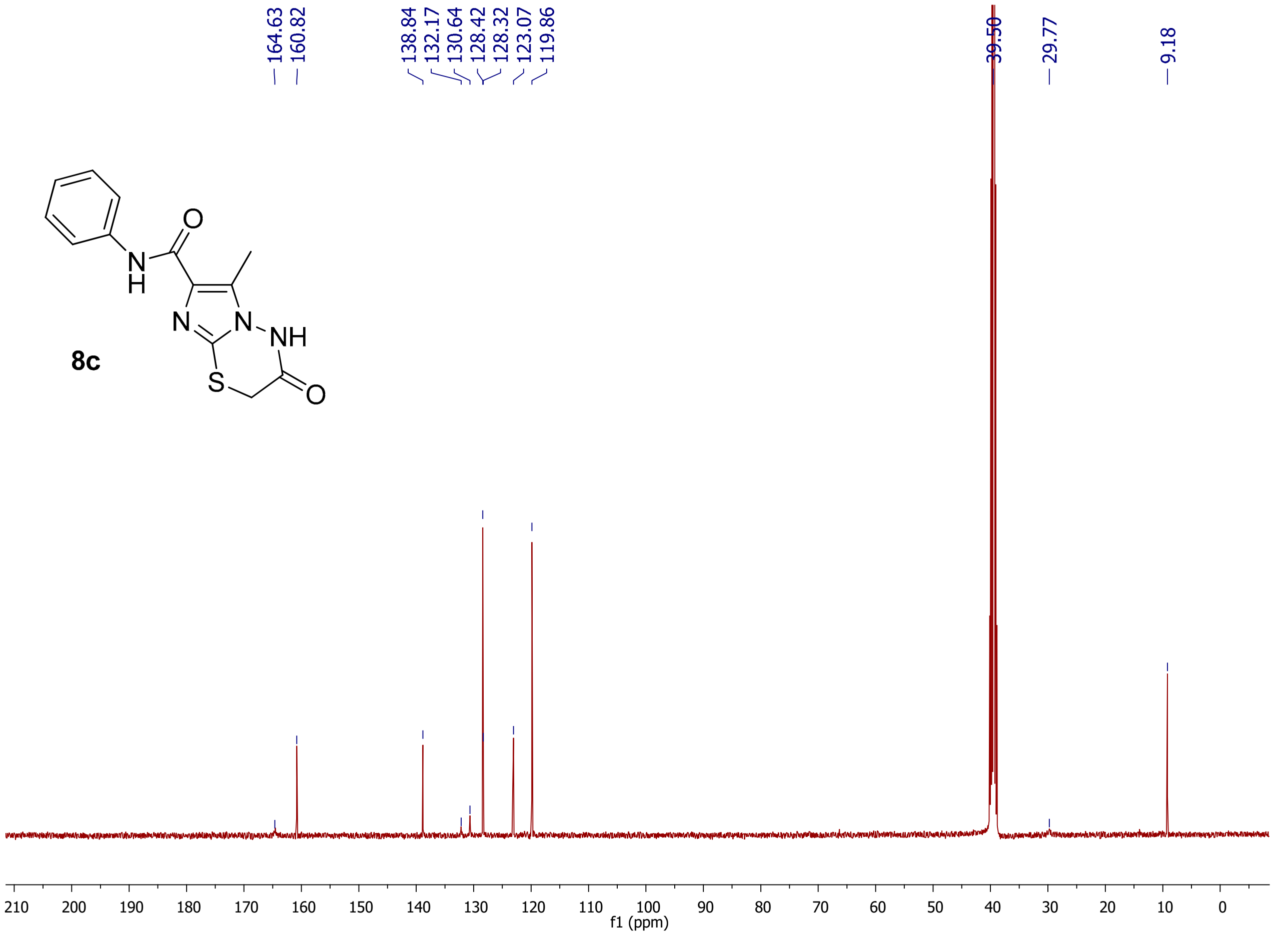
164.63
160.82
138.84
132.17
130.64
128.42
128.32
123.07
119.86
39.50
29.77
9.18
8c
O
N
H
N
N
NH
S
O
210
200
190
180
170
160
150
140
130
120
110
100
90
80
70
60
50
40
30
20
10
0
f1 (ppm)

HMQC 8c

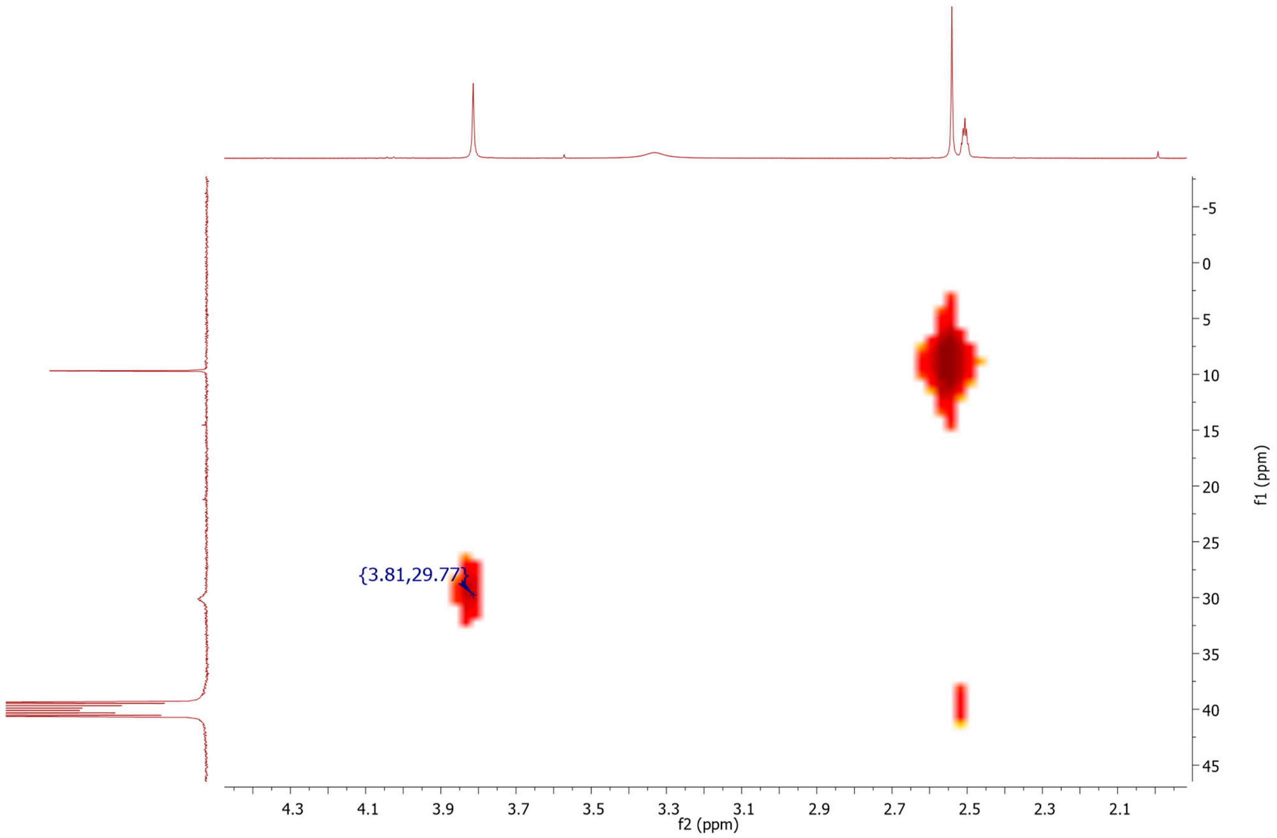

References:

1. [1] Attanasi, O. A.; Favi, G.; Filippone, P.; Perrulli, F. R.; Santeusanio, S. *Synlett* **2010**, 1859–1861, doi:10.1055/s-0030-1258108.
2. [2] Qi, L.-W.; Mao, J.-H.; Zhang, J.; Tan, B. *Nat. Chem.* **2018**, *10*, 58–64, doi:10.1038/NCHEM.2866.
